# Yolk granule fusion and microtubule aster formation regulate cortical granule translocation and exocytosis in zebrafish oocytes

**DOI:** 10.1371/journal.pbio.3002146

**Published:** 2023-06-08

**Authors:** Shayan Shamipour, Laura Hofmann, Irene Steccari, Roland Kardos, Carl-Philipp Heisenberg

**Affiliations:** 1 Institute of Science and Technology Austria, Klosterneuburg, Austria; 2 Department of Molecular Life Sciences, University of Zurich, Zurich, Switzerland; Utrecht University, NETHERLANDS

## Abstract

Dynamic reorganization of the cytoplasm is key to many core cellular processes, such as cell division, cell migration, and cell polarization. Cytoskeletal rearrangements are thought to constitute the main drivers of cytoplasmic flows and reorganization. In contrast, remarkably little is known about how dynamic changes in size and shape of cell organelles affect cytoplasmic organization. Here, we show that within the maturing zebrafish oocyte, the surface localization of exocytosis-competent cortical granules (Cgs) upon germinal vesicle breakdown (GVBD) is achieved by the combined activities of yolk granule (Yg) fusion and microtubule aster formation and translocation. We find that Cgs are moved towards the oocyte surface through radially outward cytoplasmic flows induced by Ygs fusing and compacting towards the oocyte center in response to GVBD. We further show that vesicles decorated with the small Rab GTPase Rab11, a master regulator of vesicular trafficking and exocytosis, accumulate together with Cgs at the oocyte surface. This accumulation is achieved by Rab11-positive vesicles being transported by acentrosomal microtubule asters, the formation of which is induced by the release of CyclinB/Cdk1 upon GVBD, and which display a net movement towards the oocyte surface by preferentially binding to the oocyte actin cortex. We finally demonstrate that the decoration of Cgs by Rab11 at the oocyte surface is needed for Cg exocytosis and subsequent chorion elevation, a process central in egg activation. Collectively, these findings unravel a yet unrecognized role of organelle fusion, functioning together with cytoskeletal rearrangements, in orchestrating cytoplasmic organization during oocyte maturation.

## Abstract 2

The localization and release of cortical granules at the oocyte surface constitutes a critical step in oocyte maturation in preventing polyspermy. This study shows that in zebrafish oocytes this process is driven by the concerted activities of yolk granule fusion and microtubule aster contraction.

## Introduction

Oogenesis marks the very first step in development, establishing the maternal blueprint for embryonic patterning. During this process, the oocyte grows in size by acquiring maternally provided material and completes its first meiosis to eventually become arrested in the metaphase of meiosis II until fertilization occurs [1]. Central to oogenesis is the accurate positioning of large organelles, such as the oocyte nucleus (germinal vesicle (GV)) and meiotic spindle, but also small fate-determining molecules, such as mRNAs and proteins, within the oocyte, a process fundamental for embryonic axis formation and cell fate specification [2–12]. Yet, how such positioning of cytoplasmic components is orchestrated in space and time is still only poorly understood.

Cytoplasmic organization can occur in the absence of external cues, suggesting that the cytoplasm is capable of self-organization [13,14]. Previous research has highlighted an important role for the cell cytoskeleton, and especially the microtubule and actin networks, in driving such cytoplasmic self-organization [15–17]. For instance, microtubules and the movement of motors along microtubule tracks can power cytoplasmic flows and the repositioning of microtubule asters by generating viscous drag forces to the surrounding cytoplasm [15,18–21]. Likewise, myosin II–dependent contractions of both cortical and bulk actin networks can result in large-scale actomyosin network flows, which, in turn, drag the adjacent cytoplasm via friction forces acting at their interface [16,22,23]. In addition to these motor-dependent processes, actin polymerization on the surface of organelles can drive organelle motility, thereby generating active diffusion within the bulk of the cytoplasm [23–25]. However, to what extent cellular processes other than cytoskeletal rearrangements, such as dynamic fusion and splitting of organelles, also function in cytoplasmic reorganization, remains unclear.

To tackle this question, we turned to the last stage of zebrafish oogenesis (oocyte maturation), during which cytoplasmic reorganizations are accompanied by changes in organelle shape, size, and position, preparing the oocyte for fertilization and embryonic development [26]. Zebrafish oogenesis constitutes a 5-stage process: During stages I and II, the oocyte animal-vegetal (AV) axis becomes determined through the vegetal pole localization of the Balbiani body, a membrane-less structure rich in mitochondria, proteins, and mRNAs required for the vegetal pole establishment [7,27]. Stages II and III mark the formation of both cortical granules (Cgs) and yolk granules (Ygs). Cgs are exocytosed upon fertilization to induce chorion elevation, thus preventing lethal polyspermy and protecting it against physical damage [28], while Ygs function as energy reservoirs for subsequent embryonic development [26]. Stage III oocytes remain arrested in prophase I until oocyte maturation begins. During oocyte maturation (stage IV), a multitude of transitions take place within the oocyte. For instance, the GV migrates to and breaks down at the animal pole of the oocyte, which is then followed by ooplasm, the oocyte cytoplasm, accumulating at the animal pole, forming a yolk-free blastodisc. Concomitantly, Cgs obtain exocytosis competency, thus preparing the mature oocyte/egg for fertilization [26,29–32]. How these different aspects of cytoplasmic reorganization are orchestrated and spatiotemporally controlled is still largely unknown.

Here, we show that the accumulation of Cgs at the oocyte surface and their acquisition of exocytosis competency upon GV breakdown (GVBD) are driven by the concerted activities of Yg fusion and compaction and microtubule network rearrangements, respectively. Yg fusion and compaction towards the oocyte center function in this process by inducing radially outward cytoplasmic flows that lead to the translocation and accumulation of Cgs at the oocyte surface. The microtubule network, in contrast, triggers accumulation of Rab11-positive vesicles at the oocyte surface by reorganizing into acentrosomal aster-like structures that collectively translocate towards the oocyte surface and take along Rab11-positive vesicles. Finally, the decoration of Cgs by Rab11 at the oocyte surface confers competency to Cgs to be exocytosed during mature oocyte/egg activation.

## Results

### GV breakdown triggers blastodisc formation, Yg fusion and compaction, and Cg outward flow

**Fig 1:**
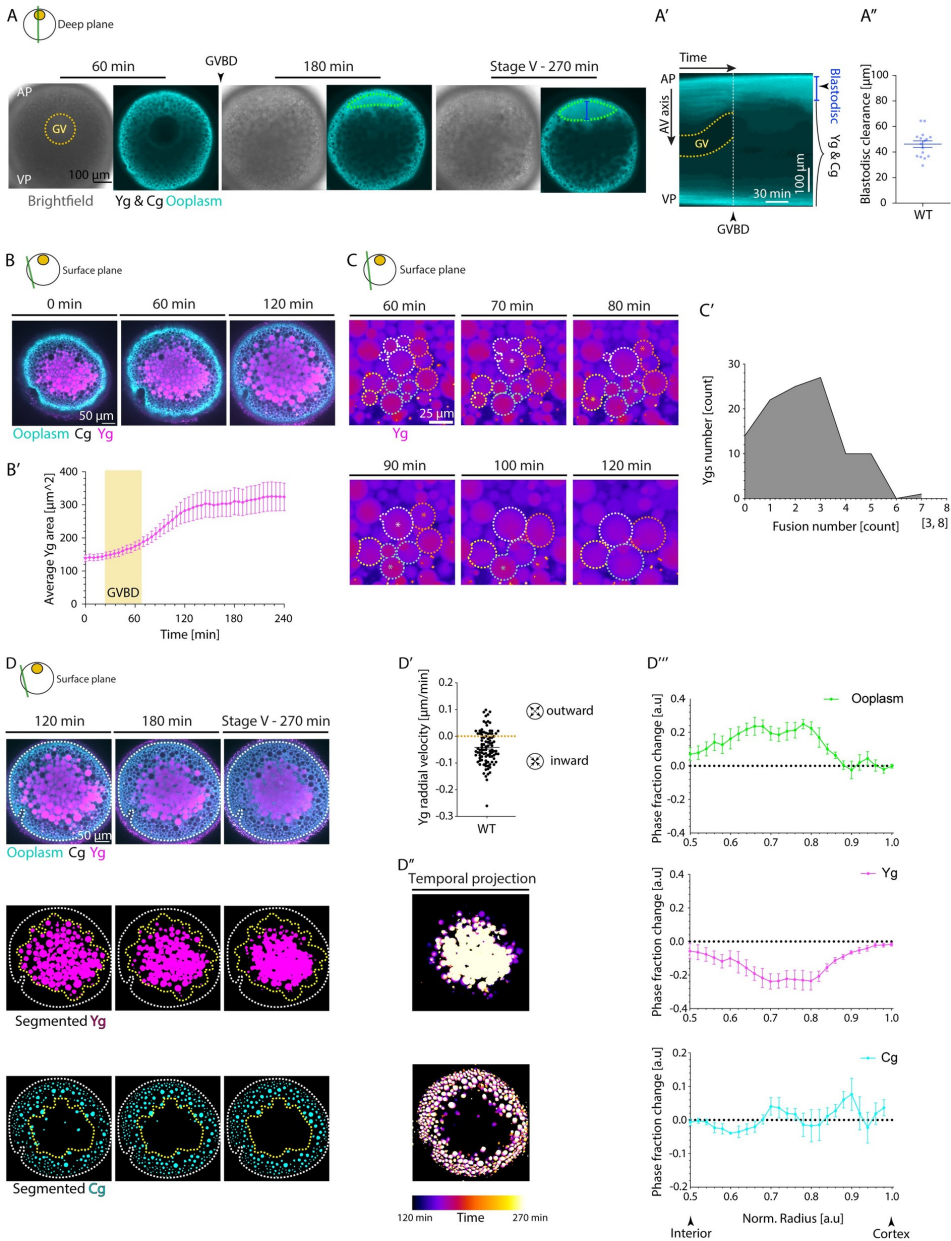
Rearrangements of the ooplasm during zebrafish oocyte maturation. (**A**) Brightfield (left) and fluorescence (right) images of stage III Tg(*hsp*:*clip170-GFP*) oocytes labeling the ooplasm during oocyte maturation at 60, 180, and 270 min after maturation induction with the DHP hormone. Dashed yellow circle highlights the GV contour, and arrowhead denotes the GVBD onset. The green ROIs indicate the blastodisc region, and blue line marks the blastodisc height as measured in Fig 1A”. Ygs and Cgs are depicted by their exclusion of ooplasmic signal. (**A’**) Kymograph acquired along the AV axis of the oocyte shown in Fig 1A as a function of time. The yellow dashed lines outline the GV contour and the dashed white line marks the time point of GVBD. The blue zone demarcates the blastodisc region. (**A”**) Blastodisc clearance, measured as the height of blastodisc at the end of the maturation process as shown in Fig 1A and 1A’, for WT oocytes (*N =* 2 experiments, *n =* 16 oocytes). See Table A in S1 Data for underlying data. (**B**) Fluorescence images of stage III Tg(*hsp*:*clip170-GFP*) oocytes labeling ooplasm (cyan) and exposed to Lysotracker to mark Yg (magenta) and Cg (black, identified by their exclusion of both Clip-170-GFP and Lysotracker) before maturation (stage III) and 60 and 120 min after maturation onset. (**B’**) Average Yg area (*N* = 3, *n* = 20) over time. Yellow box indicates the period during which GVBD takes place. See Table B in S1 Data for underlying data. (**C**) Fluorescence images of stage III oocytes exposed to Lysotracker to label Yg (magenta) at 60–120 min after maturation onset. Dashed lines with the same color demarcate the outline of Yg that will undergo fusion. Asterisks mark the time point of fusion. (**C’**) Histogram of Yg fusion events during oocyte maturation (*N =* 3, *n =* 8). See Table C in S1 Data for underlying data. (**D**) Fluorescence images of stage III Tg(*hsp*:*clip170-GFP*) oocytes labeling ooplasm (cyan) and exposed to Lysotracker to mark Yg (magenta) and Cg (black) at 120, 180, and 270 min after maturation onset (first row). Images in the bottom rows show segmented Yg and Cg obtained from the images in the first row. White dashed lines mark the oocyte outline. Yellow dashed lines denote the initial distribution of Yg in the second row and the final distribution of Cg in the third row. (**D’**) Yg radial velocity between 120 and 270 min after maturation onset (*N =* 2, *n =* 8). See Table D in S1 Data for underlying data. (**D”**) Temporal projection of segmented Yg (top) and Cg (bottom) of the oocyte shown in (**D**) between 120 and 270 min after maturation onset, illustrating the inward motion of Yg and the outward movement of Cg. (**D”’**) Changes in phase fractions for ooplasm (top, green), Yg (middle, magenta), and Cg (bottom, cyan) between 120 and 270 min after maturation onset. Normalized (norm) radii of 0.5 and 1 correspond to the oocyte interior and cortex, respectively (*N =* 3, *n =* 6). See Table E in S1 Data for underlying data. Schematics demarcate the imaging plane used for obtaining the images in each panel. Note that in panel (**A**), processes deeper within the oocyte are captured, while in panels (**B**-**D**), more superficial parts of the oocyte are captured. Error bars, SEM. AP, animal pole; AV, animal-vegetal; Cgs, cortical granules; GV, germinal vesicle; GVBD, germinal vesicle breakdown; VP, vegetal pole; WT, wild-type; Ygs, yolk granules.

To unravel the molecular, cellular, and biophysical mechanisms underlying ooplasmic reorganization during zebrafish oocyte maturation, we first analyzed how these processes occur in space and time. To this end, ovaries of female zebrafish were harvested, and stage III oocytes were isolated according to their size and ooplasmic opacity and exposed to the steroid hormone DHP triggering their maturation [26,31]. To determine which processes take place during oocyte maturation, we monitored ooplasmic reorganization in oocytes from Tg(*hsp*:*Clip170-eGFP*) females, in which the ooplasm is ubiquitously labeled by GFP (Fig 1A and S1 Movie). We found that following GV translocation to and breakdown at the animal pole of the oocyte, ooplasm accumulated at the animal pole, indicative of blastodisc formation (Fig 1A–1A”). In order to visualize which other ooplasmic rearrangements occur upon GV breakdown, we additionally marked both Ygs and Cgs within the ooplasm by exposing Tg(*hsp*:*Clip170-eGFP*) oocytes to Lysotracker dye, which exclusively labels Ygs but not Cgs, allowing us to distinguish between these different granule types (Fig 1B and S2 and S3 Movies). We further confirmed that granules not labeled by Lysotracker were indeed Cgs by showing that within the mature oocyte/egg, they colocalized with Rab11 and underwent exocytosis upon egg activation (Fig 4B), features typically associated with Cgs [28]. We found that Ygs underwent multiple fusion events during oocyte maturation, increasing their average cross-sectional area by a factor of approximately 2 (Fig 1B–1C’ and S2 Movie). To monitor how such fusion events are followed up by changes in the subcellular distribution of Ygs and Cgs within the oocyte, we focused our analysis on the second half of oocyte maturation (2 to 4.5 h after the DHP hormone addition), when the oocyte volume remained largely unchanged, thereby also removing the possible contribution of volume changes to our analysis (S1A–S1A’ Fig). By simultaneously labeling, segmenting, and following different ooplasmic components and their phase fractions during this time window, we found Ygs to further compact to the oocyte center, while both ooplasm and Cgs displayed short-range movements towards the oocyte cortex, resulting in their accumulation there (Fig 1D–1D”’ and S3 Movie). Of note, such ooplasmic rearrangements were not occurring if oocytes remained arrested at stage III in the absence of the DHP hormone (S1C Fig). Collectively, these findings suggest that oocyte maturation is temporally correlated with blastodisc formation at the AP, Yg fusion and compaction to the oocyte center, and Cg translocation towards the oocyte cortex (Figs 1 and S1D).

### Bulk actomyosin drives blastodisc expansion

Our finding that blastodisc formation is spatiotemporally linked to GV breakdown at the animal pole of the oocyte (Fig 1A) suggests that these processes might be functionally linked. Given the large size of the GV, accounting for nearly approximately 1.5% of the total stage III oocyte volume, we hypothesized that the nucleoplasm released after GV breakdown might directly result in blastodisc formation. To test this possibility, we labeled the nucleoplasm stored within the GV with fluorescently-labeled Dextran and followed its subcellular distribution after GV breakdown (S1B Fig). This showed that despite the apparent rapid diffusion of the nucleoplasm from the point of GV breakdown at the animal pole towards the vegetal pole of the oocyte, the majority of the nucleoplasm still remained at the animal pole, thereby initiating blastodisc formation (S1B–S1B” Fig). Moreover, the size of the blastodisc continued to grow after the completion of GV breakdown, rather than shrink as expected for continuous diffusion of the nucleoplasm away from the animal pole (S1B’ Fig), suggesting that mechanisms other than passive GV nucleoplasm release must be involved in blastodisc formation.

Bulk actomyosin network contraction and flows have previously been implicated in blastodisc expansion within the fertilized egg [23]. To determine whether actomyosin network contraction is also involved in the initial blastodisc formation during oocyte maturation, we exposed immature stage III oocytes to inhibitors specifically interfering with actin polymerization (Cytochalasin B (Cyto B)) and myosin II activity (para-Nitroblebbistatin (PBb)), and monitored how such treatment affects blastodisc formation. We found that in oocytes treated with 30 μg/ml Cyto B or 100 μM PBb, blastodisc expansion was strongly diminished (S2A–S2A’ Fig and S4 Movie), indicating that actomyosin network contraction is required for this process.

**Fig 2:**
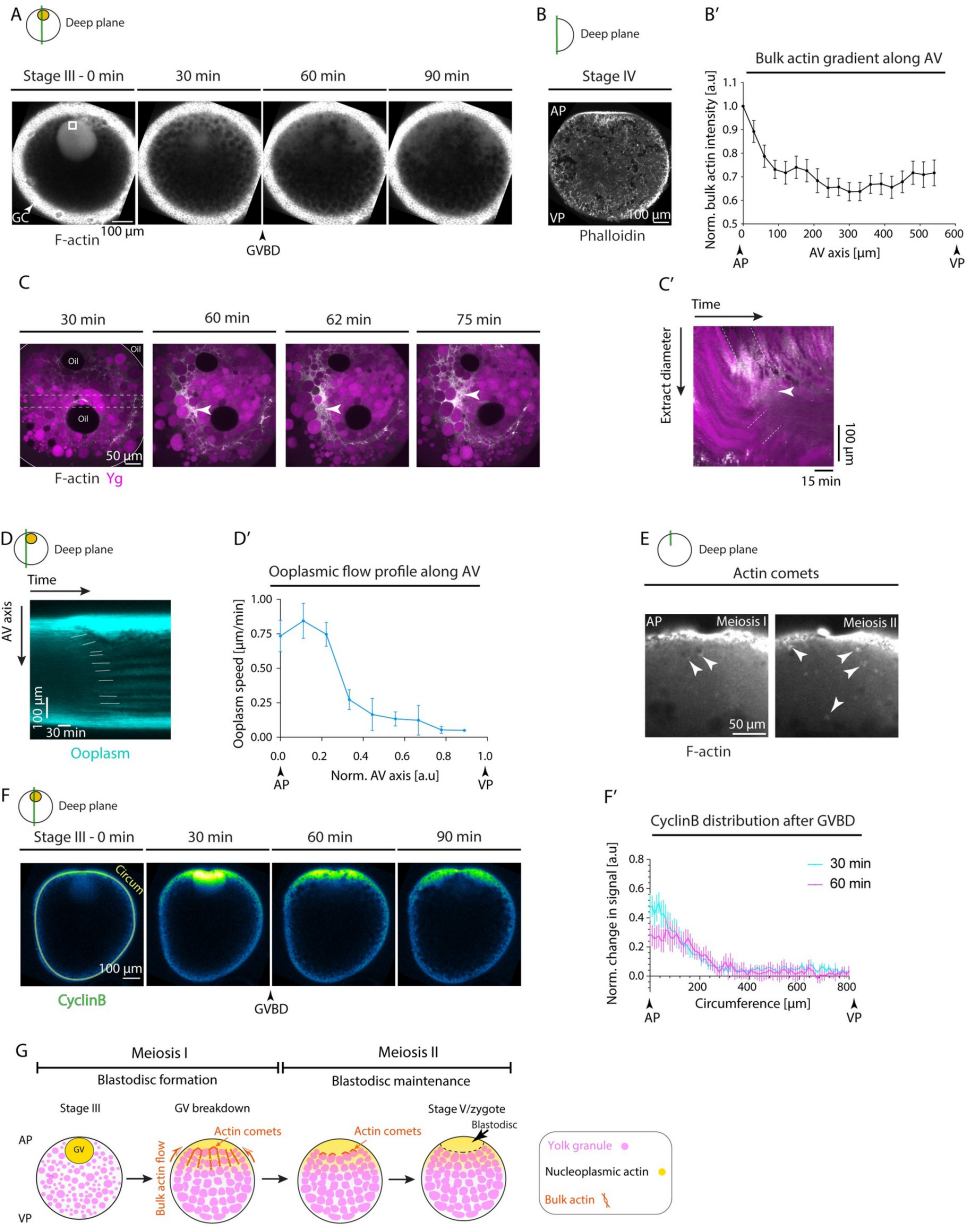
Rearrangement of the actin cytoskeleton during oocyte maturation. (**A**) Fluorescence images of stage III Tg(*actb1*:*Utr-GFP*) oocytes labeling F-actin during consecutive stages before maturation (stage III) and 30, 60, and 90 min after maturation induction with the DHP hormone. Black arrowhead denotes the GVBD onset. White arrow denotes the GCs surrounding the oocyte. The 35 μm × 35 μm white box demarcates the region for acquiring bulk actin intensity in S3B Fig. (**B**) Fluorescence image of a stage IV oocyte fixed, sectioned, and stained with Phalloidin to visualize and measure bulk F-actin gradient along the AV axis of the oocyte shown in (**B’**). (**B’**) Bulk actin intensity within cytoplasmic pockets normalized to its value at the AP along the AV axis, measured from fixed stage IV oocytes stained with phalloidin as in (**B**); (*N =* 3 experiments, *n =* 17 oocytes). See Table A in S2 Data for underlying data. (**C**) Fluorescence images of ooplasmic extract obtained from stage III Tg(*actb1*:*Utr-GFP*) oocytes labeling F-actin (gray) and exposed to Lysotracker to mark Ygs (magenta) during 30–75 min after maturation onset. The dashed box indicates the extract diameter used for acquiring the kymograph in (**C’**). The arrowheads indicate the F-actin and ooplasm enrichment. The white solid line demarcates the extract boundary. (**C’**) Kymograph acquired along the extract diameter in (**C**) as a function of time. The white dashed lines mark F-actin and ooplasmic flows, while the arrowheads indicate the F-actin and ooplasm enrichment. (**D**) Kymograph acquired along the AV axis of Tg(*hsp*:*clip170-GFP*) oocyte marking ooplasm as a function of time. White lines trace the flow of ooplasmic pockets over time. (**D’**) Ooplasmic flow profile along the AV axis, measured from the slopes of ooplasmic flow trajectories shown in (**D**). Normalized AV of 0 and 1 correspond to the animal and vegetal poles, respectively (*N =* 3, *n =* 17). See Table B in S2 Data for underlying data. (**E**) Fluorescence images of oocytes injected with 200 pg *Utrophin-GFP* mRNA to label F-actin during first and second meiosis corresponding to 135 and 170 min after maturation induction with the DHP hormone, respectively. White arrowheads mark actin comets forming within the blastodisc region on the surface of granules. (**F**) Fluorescence images of stage III oocytes injected with *CyclinB-GFP* mRNA before (stage III) and 30, 60, and 90 min after maturation onset. Arrowhead denotes the GVBD onset. The yellow line along the oocyte circumference (Circum) was used to acquire the intensity profiles plotted in (**F’**). (**F’**) Change in CyclinB signal normalized to its distribution at the time prior to GVBD, measured at 30 min (cyan) and 60 min (magenta) after GVBD along the oocyte circumference (the yellow line in F). Circumference of 0 and 800 μm correspond to the AP and VP, respectively (*N =* 2, *n =* 5). See Table C in S2 Data for underlying data. (**G**) Schematic summarizing the role of actin in ooplasmic flows and blastodisc formation. Bulk actin, initially stored within the GV, is released at the AP of the oocyte upon GVBD, thereby generating a local actin gradient. This actin gradient triggers bulk actomyosin flows towards the AP, which drags the ooplasm along, resulting in blastodisc formation. In addition, the blastodisc interface is maintained during first and second meiosis by actin comet-like structures forming on the surface of granules and—in analogy to previous observations in fertilized eggs [23]—preventing them from diffusing into the blastodisc region. Schematics in each panel demarcate the imaging plane used for obtaining the images in that panel. Error bars, SEM. AP, animal pole; AV, animal-vegetal; GC, granulosa cell; GV, germinal vesicle; GVBD, germinal vesicle breakdown; VP, vegetal pole; Ygs, yolk granules.

To determine how actomyosin network contraction functions in blastodisc formation, we analyzed dynamic changes in the intensity of F-actin during oocyte maturation using Tg(*actb1*:*Utr-GFP*) oocytes labeling F-actin. This analysis revealed that actin became enriched within the GV and was released to the surrounding ooplasm upon GV breakdown (Figs 2A and S3A and S5 Movie), where it diffused away from the animal pole towards the vegetal pole (S3A and S3A’ Fig). To examine whether these processes establish an actin gradient along the AV axis of the oocyte, we visualized F-actin distribution in sections of stage IV zebrafish oocytes that had just undergone GV breakdown using Phalloidin staining. This revealed an animal-to-vegetal bulk actin gradient with peak levels close to the animal pole of the oocyte (Fig 2B and 2B’). Such a bulk actin gradient, analogous to the situation in fertilized eggs after fertilization [23], might lead to bulk actomyosin flows directed towards the animal pole of the oocyte, which, by dragging along the ooplasm, then triggers the accumulation of ooplasm at the animal pole leading to blastodisc formation. As visualizing bulk actin flows within the opaque maturing oocyte turned out to be challenging, we prepared ooplasmic extracts from stage IV oocytes, where bulk actin and Ygs were clearly recognizable. We found that bulk actin flows within these extracts were accompanied by ooplasm flowing along and accumulating where actin aggregates were formed (Fig 2C and 2C’ and S6 Movie), suggesting that ooplasmic flows within the intact oocyte might also be driven by such bulk actin flows. Further supporting this assumption, measuring ooplasmic flows along the AV oocyte axis revealed that they occur predominantly close to the animal pole of the oocyte (Fig 2D and 2D’ and S7 Movie), where also the bulk actin gradient was detectable (Fig 2B’).

Notably, bulk actin levels ceased once the first meiosis was completed (S3A and S3A’ Fig and S8 Movie), raising questions as to the mechanisms by which the size of blastodisc is maintained during the second meiosis where no clear bulk actin was detectable anymore within the oocyte ooplasm (S3B Fig). Strikingly, we observed actin comet-like structures forming with no preferential orientation on the surface of granules located close to the blastodisc interface (Fig 2E; note that we were unable to distinguish between Ygs and Cgs in these experiments as colabelling actin and Ygs turned out to be challenging due to difficulties in detecting actin in the bulk of the ooplasm). This observation is highly reminiscent of the role of actin comet-like structures on Ygs in fertilized eggs, which promote ooplasm-Yg segregation by preventing Ygs from returning into the blastodisc region [23]. Together, our results suggest that—similar to the situation in fertilized eggs—the combined function of actin flows towards the animal pole and actin comets on the surface of granules are responsible for blastodisc formation and maintenance during oocyte maturation.

Finally, given that the changes in bulk actin dynamics were concomitant with cell cycle progression (S3A and S3A’ Fig), we asked whether CyclinB/Cdk1 complex, the key cell cycle regulator previously suggested to induce bulk actin polymerization and flows within the fertilized egg [23], might also function as an effector by which GV breakdown triggers bulk actin polymerization and flows and consequently blastodisc formation during oocyte maturation. To this end, we injected *CyclinB-GFP* mRNA into stage III oocytes (Fig 2F) and detected endogenous phosphorylated CyclinB levels by immunofluorescence (S3D Fig) to visualize its localization as a proxy for CyclinB/Cdk1 activity within the maturing oocyte [33]. We found CyclinB to be highly enriched within the GV of stage III oocytes and then released to the surrounding ooplasm upon GV breakdown (Figs 2F and S3D and S9 Movie). Importantly, the release of CyclinB led to a spatially restricted gradient of CyclinB at the oocyte animal pole, which persisted for more than 1 h after GV breakdown (Fig 2F’), highly reminiscent of the gradients observed for the nucleoplasm (S1B–S1B” Fig) and bulk actin (Fig 2B and 2B’) following GV breakdown. In line with the role of CyclinB in bulk actin polymerization, their dynamics were also temporally coordinated, both peaking at the GVBD onset (S3B and S3C Fig). Moreover, inhibiting CyclinB synthesis by exposing stage IV oocytes undergoing maturation to 700 μM Cycloheximide resulted in decreased ooplasmic flows and blastodisc formation (S2A and S2A’ Fig and S4 Movie). This suggests that the activation and release of CyclinB/Cdk1 at the animal pole of the oocyte drives bulk actin polymerization, which, in turn, triggers bulk actin and ooplasmic flows leading to blastodisc formation (Fig 2G).

### Microtubule network forms asters upon GV breakdown

**Fig 3:**
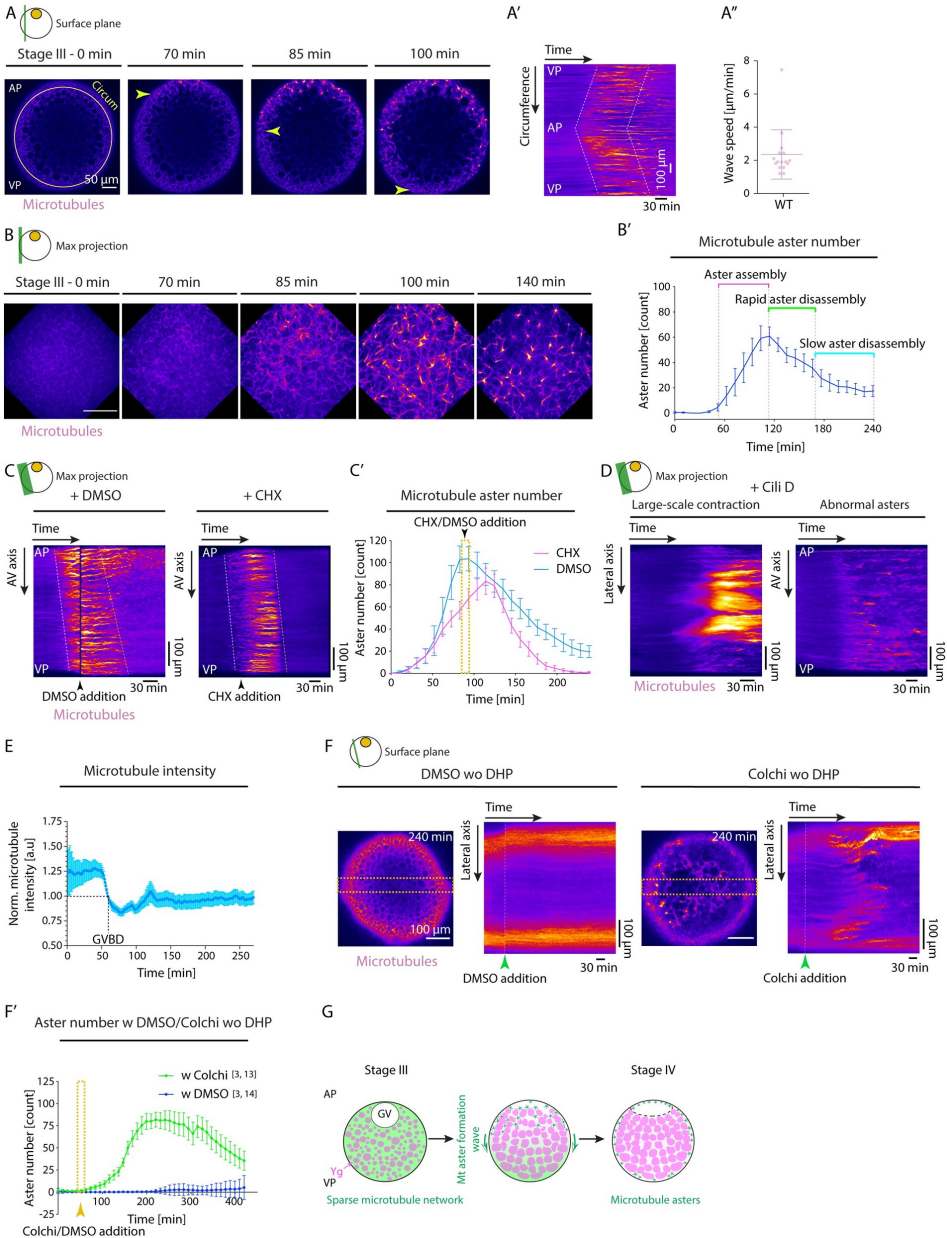
Rearrangement of the microtubule cytoskeleton during oocyte maturation. (**A**) Fluorescence images of stage III Tg(*Xla*.*Eef1a1*:*dclk2a-GFP*) oocytes labeling microtubules before (stage III) and 70, 85, and 100 min after maturation onset. Arrowheads mark the propagating front of the microtubule aster formation wave. The circumferential (Circum) line in the first panel indicates the region used for acquiring the kymograph in (**A’**). (**A’**) Kymograph acquired along the circumference of the oocyte shown in (**A**) as a function of time. Dashed lines mark the leading and trailing edges of the microtubule aster formation wave. (**A”**) Speed of microtubule aster formation wave along the oocyte circumference, measured from kymographs as shown in (**A’**); *N =* 9 experiments, *n =* 16 oocytes. See Table A in S3 Data for underlying data. (**B**) Maximum fluorescence intensity projection of high-resolution images of stage III Tg(*Xla*.*Eef1a1*:*dclk2a-GFP*) oocytes labeling microtubules during consecutive stages before (stage III) and 70, 85, 100, and 140 min after maturation onset. (**B’**) Microtubule aster number as a function of time. Aster assembly during the second hour after maturation onset is followed by initially rapid and then slower disassembly phases (*N* = 2, *n* = 8). See Table B in S3 Data for underlying data. (**C**) Kymographs acquired along the AV axis of Tg(*Xla*.*Eef1a1*:*dclk2a-GFP*) oocytes exposed to DMSO (control, left) or 700 μM CHX (right) as a function of time. Arrowheads mark the time point of oocyte exposure to DMSO/CHX. Dashed lines mark the leading and trailing edges of the microtubule aster formation wave. (**C’**) Microtubule aster number as a function of time for Tg(*Xla*.*Eef1a1*:*dclk2a-GFP*) oocytes exposed to DMSO (cyan, *N =* 2, *n =* 8) or CHX (magenta, *N* = 2, *n* = 9). The yellow box indicates the time window of oocyte exposure to DMSO or CHX in the respective experiments. See Table C in S3 Data for underlying data. (**D**) Kymographs acquired along the lateral (left) and AV (right) axis of exemplary Tg(*Xla*.*Eef1a1*:*dclk2a-GFP*) oocytes exposed to Cili D as a function of time demonstrating large-scale contractions and abnormal aster formation, respectively. (**E**) Microtubule intensity measured in a 50 pix-wide region at the blastodisc in the vicinity of the GV over time normalized to intensity values at GVBD onset (*N =* 2, *n =* 9). See Table D in S3 Data for underlying data. (**F**) Fluorescent images of stage III Tg(*Xla*.*Eef1a1*:*dclk2a-GFP*) oocytes exposed to DMSO (control, left) or Colchi (right) for 240 min in the absence of DHP (immature oocyte). The orange dashed boxes denote the lateral regions used for obtaining the kymographs shown on the right. The panels on the right indicate the kymographs of microtubule intensity along the lateral axis of the oocytes shown on the left as a function of time. The green arrowheads denote the time point of oocyte exposure to DMSO or Colchi. (**F’**) Microtubule aster number as a function of time for oocytes exposed to DMSO (blue, *N* = 3, *n* = 14) or Colchi (green, *N* = 3, *n* = 13) without DHP (immature oocytes). The arrowhead denotes the time point of oocyte exposure to DMSO or Colchi. See Table E in S3 Data for underlying data. (**G**) Schematic summarizing the microtubule network transformation taking place during oocyte maturation. A uniform microtubule network encompassing the ooplasm of the immature oocytes reorganizes into numerous microtubule asters in a wave-like fashion from the AP to the VP of the oocyte. This network transformation relies on the partial depolymerization of the microtubule network initiated by GVBD at the AP of the oocyte. Schematics in each panel demarcate the imaging plane used for obtaining the images in that panel. Error bars, SEM. AP, animal pole; AV, animal-vegetal; CHX, Cycloheximide; Cili D, Ciliobrevin D; Colchi, Colchicine; GV, germinal vesicle; GVBD, germinal vesicle breakdown; VP, vegetal pole; WT, wild-type; Ygs, yolk granules.

To determine whether other cytoskeletal elements, and in particular microtubules, might also have a function in ooplasmic reorganization during oocyte maturation, we exposed immature stage III oocytes to inhibitors specifically interfering with microtubule assembly/disassembly (Colchicine and Taxol) and monitored how such treatment affects ooplasmic reorganization and oocyte maturation. We found that in oocytes treated with 200 μM of Colchicine, blastodisc formation was largely unaffected (S2A and S2A’ Fig and S4 Movie), suggesting that microtubules might be dispensable for this process. Interestingly, however, both Colchicine- and Taxol-treated oocytes displayed strongly reduced chorion elevation, a process previously shown to be mediated by the release of Cgs at the oocyte surface ([28,32]; S2B and S2B’ Fig). To understand whether and how microtubules might be involved in Cg relocalization and/or exocytosis during oocyte maturation, we first monitored how the microtubule cytoskeleton changes during oocyte maturation in Tg(*XlEef1a1*:*dclk2-GFP*) oocytes labeling microtubules. We found microtubules to be uniformly distributed throughout the ooplasm of stage III oocytes (Fig 3A). This microtubule network, however, transformed drastically upon GV breakdown with numerous bright foci of microtubules appearing in the bulk of the ooplasm (Fig 3A and S10 Movie). Closer examination of these foci revealed that they resembled microtubule aster-like structures undergoing dynamic fusion and splitting over time (Fig 3B and S11 Movie), the number of which increased shortly after GV breakdown and then dropped again (Fig 3B’). These consecutive phases of aster formation and disappearance gave rise to a microtubule transformation wave propagating from the animal to the vegetal pole of the oocyte, where asters were forming at the leading edge and dissolving again at the trailing edge (Fig 3A’–3A”).

We next asked what signals might trigger this microtubule transformation wave. Given that the microtubule transformation wave was initiated upon GV breakdown and that the cell cycle regulator CyclinB/Cdk1 complex, stored within GV (Figs 2F and S3D), has previously been found to trigger microtubule reorganization [34–37], we hypothesized that the release of CyclinB/Cdk1 at the animal pole upon GV breakdown and its diffusion towards the vegetal pole of the oocyte might be involved in this process. To test this possibility, we exposed stage IV oocytes undergoing maturation to 700 μM Cycloheximide inhibiting the synthesis of CyclinB or to 250 μM of the specific Cdk1 inhibitor Dinaciclib (Figs 3C and S4A). Strikingly, microtubule asters prematurely disappeared in oocytes exposed to Cycloheximide or Dinaciclib, leading to a more homogeneous distribution of microtubules reminiscent of the situation in immature oocytes before GV breakdown (Figs 3C and 3C’ and S4A and S12 Movie). This suggests that CyclinB/Cdk1 activation and release upon GV breakdown drives the observed microtubule transformation wave. Importantly, blocking F-actin polymerization by treating oocytes with 20 μg/ml Cyto B did not interfere with microtubule aster formation, suggesting that these mechanisms are distinct at the molecular level (S4B Fig).

### Partial microtubule depolymerization drives microtubule aster formation during oocyte maturation

The formation of aster-like acentrosomal microtubule structures in *Xenopus* egg extracts has previously been shown to rely on the activity of microtubule motor dynein, clustering microtubule minus ends to the aster center [38]. Hence, we asked whether dynein motors might also be involved in mediating the effect of the CyclinB/Cdk1 complex on microtubule aster formation during zebrafish oocyte maturation. To this end, we exposed stage III oocytes to 75 μM of the dynein inhibitor Ciliobrevin D [15]. Microtubule asters of Ciliobrevin D–treated oocytes failed to fully contract and generated abnormal asters and/or dense networks remaining connected across large distances within the oocyte (Figs 3D and S4C and S13 Movie), suggesting that dynein motor activity is needed for proper microtubule aster formation.

To further determine whether dynein motor activity needs to be high for microtubules forming asters, as previously suggested [36], we estimated the motor force regime underlying this microtubule network contraction/aster formation process during oocyte maturation. To this end, we measured microtubule contraction length scales by identifying the domain that gives rise to each microtubule cluster detectable at the final time point of contraction and determined the size of the first and second biggest domains (*ξ*_1_ and *ξ*_2_), as used in percolation theory to describe the mode of network contraction [39]. In the local contraction regime, *ξ*_1_ and *ξ*_2_ are of similar magnitude, while for large-scale contractions, *ξ*_1_ becomes close to the system size at the expense of *ξ*_2_ shrinking in size. To explore the *ξ*_1_ versus *ξ*_2_ space more systematically, we performed a network contraction analysis in oocytes exposed to DMSO (control) or 75 μM Ciliobrevin D to inhibit dynein motor activity. This analysis indicated that control oocytes exhibit local contractions (*ξ*_1_≈*ξ*_2_; S4D and S4D’ Fig), while 40% of maturing oocytes exposed to 75 μM Ciliobrevin D exhibited large-scale network contractions (*ξ*_1_>*ξ*_2_; S4D Fig), where the size of the biggest cluster reaches close to the system size (*ξ*_1_ ≈ 200 μm). Based on predictions from active gel contractility [39], such local network contractions, and ultimately the formation of small-sized asters, in control, but not dynein-inhibited oocytes, suggests that the dynein motor activity levels are high within maturing oocytes.

Given that microtubule aster formation is thought to be driven by increasing the ratio of microtubule motors to microtubule number [40], we further asked whether a reduction in microtubule number upon GV breakdown, and thus an increase in the ratio of microtubule motors to microtubule number, might lead to the observed aster formation of the microtubule network. To this end, we analyzed whether the total amount of polymerized microtubules changes upon GV breakdown by monitoring intensity changes in the vicinity of the GV in Tg(*XlEef1a1*:*dclk2-GFP*) oocytes labeling microtubules (Figs 3E and S4E). This analysis showed that the total amount of polymerized microtubules decreased up to approximately 30% of its initial levels just as the first microtubule asters appeared in the ooplasm (Fig 3E). To determine whether such depolymerization of microtubules would be sufficient to trigger this transformation, we treated immature stage III oocytes, still displaying uniform microtubule distribution, to 300 μM of the microtubule depolymerizing drug Colchicine or DMSO as control (Fig 3F) and analyzed resultant changes in microtubule network organization. Remarkably, partial depolymerization of microtubules in Colchicine-treated stage III oocytes led to the premature formation of numerous microtubule asters displaying extensive fusion and splitting dynamics, while the microtubule network of DMSO-treated control oocytes remained unchanged (Fig 3F and 3F’ and S14 Movie). Interestingly, the network contraction analysis of the microtubule asters forming in Colchicine-treated immature oocytes revealed small contraction length scales, indicative of the high dynein activity present already within the immature oocytes before GV breakdown (S4D-S4D’ Fig). This suggests that the partial depolymerization of microtubules, rather than increased dynein motor activity, upon GV breakdown is responsible for the microtubule network transformations observed during oocyte maturation.

Collectively, these findings suggest a model where the microtubule network in early stage III oocytes is in a “jammed” configuration that cannot reorganize despite its high motor activity. The partial disassembly of this network upon GV breakdown will, in turn, “unjam” the network by increasing the ratio of microtubule motors to microtubules, eventually leading to microtubule aster formation (Fig 3G).

### Microtubule asters ensure proper chorion elevation upon activation

**Fig 4:**
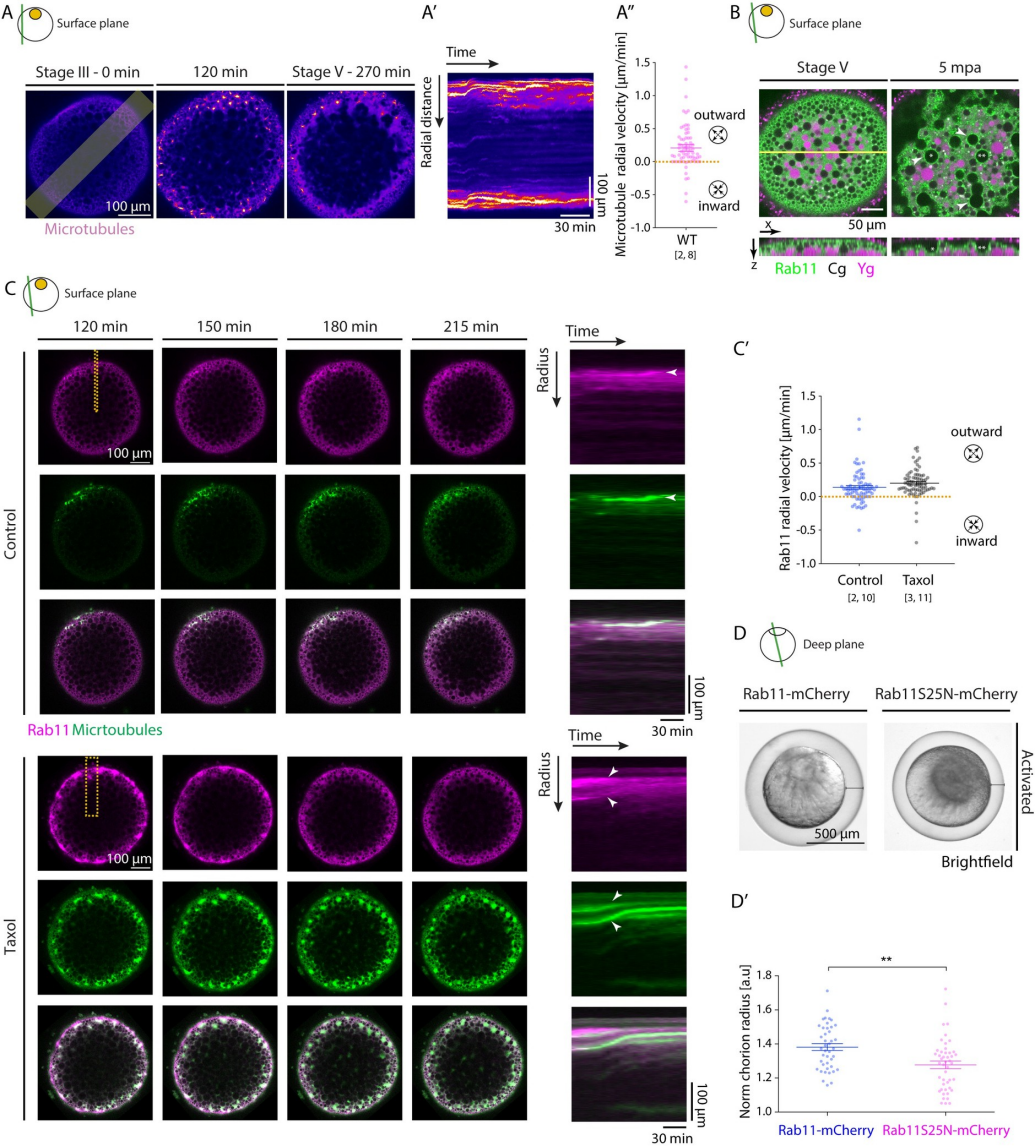
Rab11 vesicles move towards the oocyte cortex together with microtubule asters. (**A**) Left: Fluorescence images of stage III Tg(*Xla*.*Eef1a1*:*dclk2a-GFP*) oocytes labeling microtubules before (stage III) and 120 and 270 min after maturation onset. The lateral line indicates the region used for acquiring the kymograph in (**A’**). (**A’**) Kymograph of microtubule intensity along the lateral axis of the oocyte shown in (**A**) as a function of time. (**A”**) Microtubule aster radial velocity during maturation onset (*N =* 2 experiments, *n =* 8 oocytes). See Table A in S4 Data for underlying data. (**B**) Fluorescence images of Tg(*actb2*:*Rab11a-NeonGreen*) oocytes marking Rab11-positive vesicles (green) and exposed to Lysotracker to label Ygs (magenta) at stage V (mature oocyte/egg, left) and 5 min after activation (mpa) with E3 medium (right). Cgs (black) are identified by their exclusion of Lysotracker and the ooplasmic signal. The yellow line indicates the region used for displaying the orthogonal view (bottom images). Asterisks mark exemplary Cg undergoing exocytosis, and arrowheads denote the localization of Rab11 on Cg prior to/during their exocytosis. (**C**) Left: Fluorescence images of stage III Tg(*actb2*:*Rab11a-NeonGreen*) oocytes marking Rab11-positive vesicles (magenta, top rows) and injected with 400 pg of *DCLK-mKO2* mRNA to label microtubules (green, middle rows) 120, 150, 180, and 215 min after maturation onset in control oocytes (WT, top panels) or oocytes exposed to 50 μM Taxol (bottom panels). Overlaid images are shown in the bottom rows. Dashed lines indicate the regions used for acquiring kymographs on the right. Right: Kymographs acquired along the marked area of the oocytes shown on the left as a function of time. Arrowheads point at exemplary Rab11-positive vesicles or microtubule asters moving towards the cortex. (**C’**) Rab11 radial velocity during maturation onset for control oocytes (left, *N =* 2, *n =* 10) and oocytes exposed to 50 μM Taxol (right, *N* = 3, *n* = 11). See Table B in S4 Data for underlying data. (**D**) Brightfield images of oocytes injected with 350 pg of *Rab11-mcherry* (left) or *Rab11S25N-mCherry* (right; DN) mRNA, induced to undergo oocyte maturation for 270 min and activated consequently by exposure to E3 medium for 30 min. Black lines demarcate the distance between the egg and its overlaying chorion. (**D’**) Chorion elevation, measured as chorion diameter normalized to the oocyte diameter, of oocytes injected with 350 pg of *Rab11-mcherry* (blue, control, *N* = 3, *n* = 42) or *Rab11S25N-mCherry* (magenta, *N* = 3, *n* = 46) mRNA. See Table C in S4 Data for underlying data. Schematics in each panel demarcate the imaging plane used for obtaining the images in that panel. Error bars, SEM. Mann–Whitney test, **p = 0.001. Cgs, cortical granules; DN, dominant negative; WT, wild-type; Ygs, yolk granules.

To determine whether and how the transformation of the microtubule network into acentrosomal asters is linked to its apparent requirement for chorion elevation, as suggested by our microtubule interference experiments (S2B and S2B’ Fig), we analyzed the spatiotemporal dynamics of microtubule aster formation relative to the reorganization of Ygs, Cgs, and ooplasm. This analysis revealed that microtubule asters, while travelling as a wave from the animal to the vegetal pole of the oocyte, exhibited a radially outward-directed flow from the center to the surface of the oocyte, leading to microtubule aster accumulation in cortical regions of the oocyte (Fig 4A–4A” and S15 Movie). Notably, the outward flow of microtubule asters was also observed in immature oocytes treated with Colchicine to trigger premature microtubule depolymerization-mediated aster formation (Fig 3F), suggesting that this outward flow is due to some inherent radial polarity of the oocyte. Consistent with this possibility, the subcellular localization of the microtubule-cortex anchoring proteins Dynein and Numa [41], visualized by immunofluorescence, displayed strong cortical accumulation all around the oocyte periphery (S4F and S4F’ Fig), pointing at the possibility that higher levels of dynein at the cortex might trigger the outward movement of microtubule asters by preferentially anchoring microtubules to the oocyte cortex [42]. In line with this assumption, in oocytes exposed to Ciliobrevin D, abnormal aster formation was accompanied by reduced movement of these aster-like structures towards the oocyte cortex (S4G and S4G’ Fig).

Given the similarity in the radially outward-directed movement of both microtubule asters and Cgs during oocyte maturation (Figs 1D” and 4A’), we asked whether microtubules might drag Cgs towards the cortex where their exocytosis is needed for chorion elevation. However, interfering with microtubule aster formation by treating stage III oocytes with 200 μM Colchicine did not affect Cg accumulation at the oocyte cortex (S5A and S5A’ Fig), suggesting that microtubules do not function in chorion elevation by transporting Cgs towards the oocyte cortex.

Alternatively, microtubule aster formation and translocation towards the oocyte cortex might be required for chorion elevation by regulating Cg exocytosis. The Rab family of proteins regulates various aspects of cellular trafficking and has been implicated in Cg exocytosis in various animal species [43–45]. In particular, Rab11 has previously been shown to localize to the surface of Cgs in both zebrafish and *C*. *elegans* oocytes [28,44] and to be required for the synchronous secretion of Cgs upon fertilization in *C*.*elegans* [44]. We thus asked whether the outward moving microtubule asters might be involved in chorion elevation by transporting Rab11-positive vesicles towards the oocyte cortex, where Cgs reside, thereby facilitating their exocytosis by decorating Cgs with Rab11. To test this possibility, we generated Tg(*actb1*:*Rab11a-NeonGreen*) animals, allowing us to visualize Rab11 dynamics within the maturing oocyte. Consistent with previous observations, we found Rab11 to colocalize with Cgs at the oocyte surface upon egg activation (Fig 4B and S16 Movie). Interestingly, we also found that Rab11-positive vesicles displayed outward flows together with microtubule asters during oocyte maturation and eventually colocalized with Cgs at the cortex of mature oocytes/eggs (Fig 4C and S17 Movie). To determine whether microtubules are involved in this translocation of Rab11-positive vesicles to the oocyte cortex, we analyzed whether Rab11-positive vesicle distribution changes when microtubule aster formation is altered in oocytes. To interfere with microtubule dynamics, we treated oocytes with 200 μM of the microtubule depolymerizing drug Colchicine and found a strongly reduced density of Rab11-positive vesicles at the cortex of treated mature oocytes/eggs (S6A and S6A’ Fig; [36]). In contrast, stabilizing microtubules with 50 μM Taxol led to the formation of enlarged microtubule aster-like structures, accompanied by Rab11-positive vesicles aggregating on and moving together with these enlarged microtubule asters towards the oocyte surface (Fig 4C and 4C’ and S18 Movie). In line with a critical function of microtubules in Rab11 translocation to the oocyte surface and decoration of Cgs, we found both Cg exocytosis and chorion elevation to be compromised in both Colchicine- or Taxol-treated mature oocytes/eggs (S6B, S6B’ and S2B-S2B’ Figs). Finally, to investigate whether Rab11 is indeed required for Cg exocytosis, and thus chorion elevation, we expressed a dominant-negative variant of Rab11, Rab11^S25N^ [46], to block Rab11 activity during oocyte maturation (Fig 4D). Strikingly, we found that overexpression of Rab11^S25N^ led to strongly reduced chorion elevation in activated oocytes/eggs (Fig 4D’), suggesting that Rab11 activity is required for this process. In contrast, the overexpression of Rab11^S25N^ had no clearly recognizable effects on microtubule aster formation and/or Cg relocalization (S6C and S6D Fig). Taken together, these results indicate that microtubule asters, by moving towards the cortex upon GV breakdown, take along Rab11-positive vesicles, and that this cortical translocation of Rab11-positive vesicles is required for the decoration of Cgs at the cortex with Rab11 and, thus, Cg exocytosis.

### Yolk granule fusion triggers cytoplasmic flows transporting cortical granules towards the oocyte cortex

**Fig 5:**
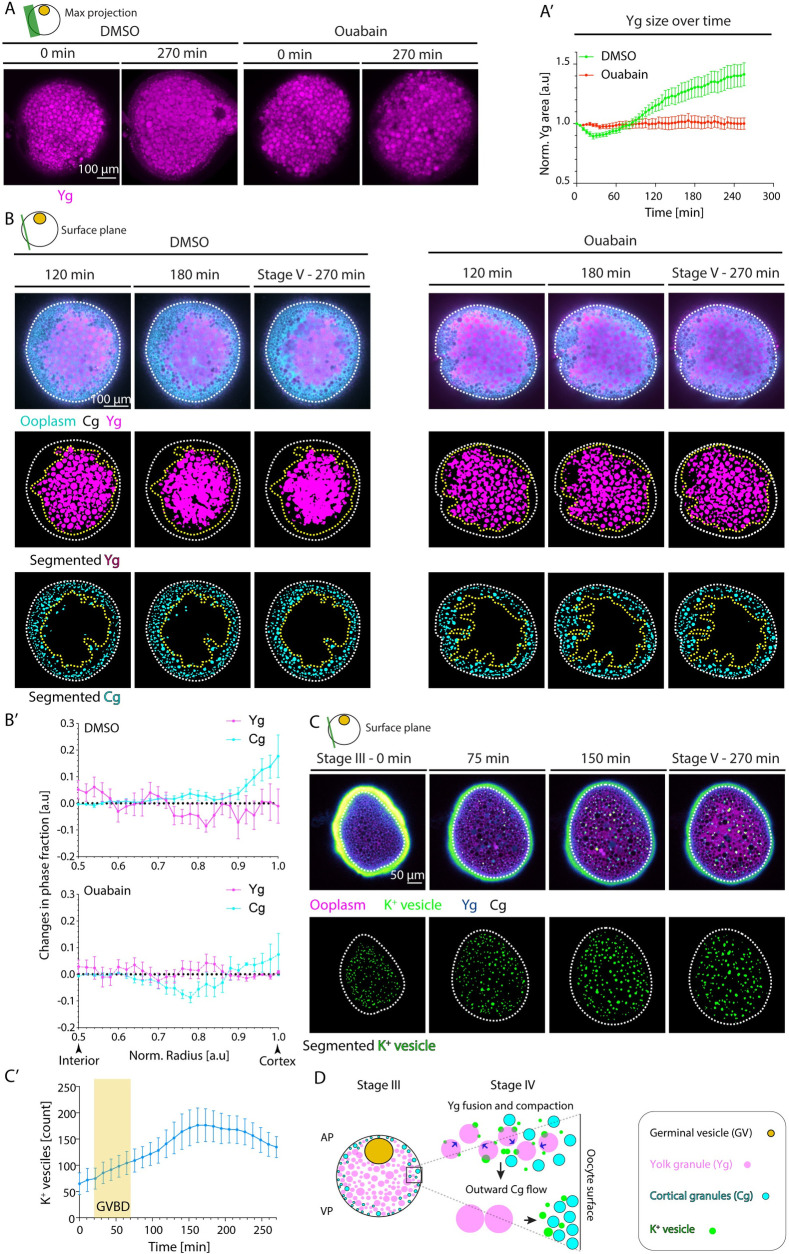
Yg fusion leads to Cg translocation towards the oocyte cortex. (**A**) Maximum fluorescence intensity projection of oocytes exposed to DMSO (left) or Ouabain (right) and Lysotracker for labeling Ygs before (stage III) and 270 min after maturation onset. (**A’**) Average Yg cross-sectional area normalized to its value at stage III as a function of time during oocyte maturation for oocytes exposed to DMSO (green, *N =* 3 experiments, *n =* 15 oocytes) or Ouabain (red, *N =* 3, *n =* 14). See Table A in S5 Data for underlying data. (**B**) Fluorescence images of DMSO-treated (left panels) and Ouabain-treated (right panels) stage III Tg(*hsp*:*clip170-GFP*) oocytes labeling ooplasm (cyan) and exposed to Lysotracker to mark Yg (magenta) and Cgs (black, identified by their exclusion of both Clip-170-GFP and Lysotracker) at 120, 180, and 270 min after maturation onset (top rows). Images in the middle and bottom rows show segmented Yg and Cg, respectively, obtained from the images in the first rows. White dashed lines mark the oocyte outline. Yellow dashed lines denote the initial distribution of Yg in the middle rows and the final distribution of Cg in the bottom rows. (**B’**) Changes in phase fractions of Yg (magenta) and Cg (cyan) for oocytes exposed to DMSO (top, *N* = 3, *n* = 9) or Ouabain (bottom, *N* = 2, *n* = 8) between 120 and 270 min after maturation onset. Normalized (norm) radii of 0.5 and 1 correspond to the oocyte interior and cortex, respectively. See Table B in S5 Data for underlying data. (**C**) Top row: Fluorescence images of stage III oocytes injected with *K*^+^ indicator (*K*^+^-Green, green) and Dextran Alexa Fluor 647 to mark ooplasm (magenta) before (stage III) and 75, 150, and 270 min after maturation onset. Cgs (black) are identified by their exclusion of both Dextran and *K*^+^-Green. The vesicles enriched with *K*^+^ fused with each other and with Yg, thereby increasing their internal *K*^+^ concentration and hence becoming dark blue (in Green-Fire-Blue Lookup Table). Bottom row: Segmented *K*^+^ vesicles identified from the images in the top row. White dashed lines mark the oocyte outline. (**C’**) Number of *K*^+^ vesicles in superficial stacks of the oocyte as in (**C**) as a function of time (*N* = 2, *n* = 8). Yellow box indicates the period during which GVBD takes place. See Table C in S5 Data for underlying data. **(D)** Schematic summarizing the role of Yg fusion in ooplasmic reorganizations during zebrafish oocyte maturation. GVBD leads to Yg fusion and compaction by triggering a *Na*^+^/*K*^+^ ATPase-dependent increase in the concentration of *K*^+^ within Yg. The fusion and compaction of Yg to the oocyte center, in turn, leads to ooplasm flows directed towards the oocyte surface. These ooplasmic flows, in turn, carry along Cg, thereby moving them closer to the cortex. Schematics in each panel demarcate the imaging plane used for obtaining the images in that panel. Error bars, SEM. Cg, cortical granule; GVBD, germinal vesicle breakdown; Yg, yolk granule.

Questions remain as to the mechanisms underlying the relocalization of Cgs towards the oocyte cortex since neither the inhibition of myosin II activity nor the depolymerization of microtubules affected Cg relocalization (S5A and S5A’ Fig and S19 Movie), arguing against a direct involvement of these cytoskeletal networks (or at least the specific components tested in our experiments) in this process. To identify such mechanism(s), we performed a detailed spatiotemporal analysis of Cg translocation towards the oocyte surface. This showed that in immature stage III oocytes, Cgs were distributed in between Ygs (Fig 1D). However, as oocyte maturation proceeded and GV breakdown occurred at the oocyte animal pole, Ygs underwent extensive fusion and compaction concomitant with Cgs translocation towards the oocyte cortex (Fig 1D” and 1D”’). Generally, the compaction of a compressible material embedded within an incompressible fluid is expected to drive outward fluid flows due to volume conservation. Therefore, we postulated that Yg fusion and compaction might result in outward ooplasmic flows, which, in turn, take along Cgs, thereby translocating them to the oocyte surface. To directly test this possibility, we treated oocytes with 100 μM Ouabain, a known inhibitor of *Na*^+^/*K*^+^ ATPase pumps [47], as an increase in intracellular K^+^ levels has previously been suggested to induce Yg fusion in other teleosts [1,48]. In oocytes treated with Ouabain, the oocyte remained opaque and Ygs largely failed to fuse and compact towards the oocyte center (Figs 5A, 5A’, S7A and S7B and S20 Movie; [48]). Moreover, Cg translocation to the cortex was strongly reduced (Fig 5B and 5B’ and S19 and S21 Movies), suggesting that Yg fusion and compaction are required for Cg translocation to the oocyte surface. Notably, we also observed that not only Cg translocation but also blastodisc formation was reduced in Ouabain-treated oocytes (S7C and S7C’ Fig), suggesting that Yg fusion and resultant outward cytoplasmic flows also contribute to blastodisc formation. In line with such a function, we found that Yg fusion was more pronounced at the animal pole of the oocyte, where the blastodisc is forming (S7D and S7D’ Fig). Importantly, these different effects of Ouabain treatment were not due to Ouabain affecting GV breakdown and, thus, cell cycle progression and its associated actin and microtubule cytoskeletal rearrangements, or the deposition of Rab11-positive vesicles at the surface of mature oocytes/eggs, as all of these processes still occurred in the presence of Ouabain treatment (S7E–S7G Fig). Taken together, these results suggest that Yg fusion and its associated radially outward-directed ooplasmic flows drive the translocation of Cg to the oocyte cortex and contribute to blastodisc formation at the oocyte animal pole.

Finally, we asked what signals might trigger Yg fusion. Given that an increase in intracellular *K*^+^ levels has previously been proposed to trigger YG fusion in Sea Bass oocytes [48], we monitored dynamic changes in intracellular *K*^+^ levels during oocyte maturation by injecting a *K*^+^ indicator (*K*^+^-Green) into the immature stage III oocyte. Strikingly, we found *K*^+^ to become enriched in small vesicles, which increased in number upon GV breakdown and underwent extensive fusion with each other and Ygs, ultimately increasing *K*^+^ levels inside Ygs undergoing fusion (Fig 5C and 5C’). In contrast, no such spatiotemporal changes in *K*^+^ levels upon GV breakdown were detected in Ouabain-treated oocytes defective in Yg fusion (S7H and S7H’ Fig and S22 Movie). Given that Ouabain blocks *Na*^+^/*K*^+^ ATPase pumps, this suggests that GV breakdown leads to Yg fusion by triggering a *Na*^+^/*K*^+^ ATPase-dependent increase in the concentration of *K*^+^ within Ygs. This Yg fusion and compaction to the oocyte center, in turn, triggers radially outward-directed ooplasmic flows, which carry along and position Cgs at the oocyte cortex (Fig 5D).

## Discussion

Our study provides novel insight into both the processes underlying oocyte maturation and the general mechanisms by which cytoplasmic reorganization is achieved within cells. Cgs are Golgi-derived secretory vesicles, which localize to the cortex of the mature oocytes/eggs to undergo exocytosis upon fertilization and induce chorion elevation/modification, thus preventing the entry of additional sperm into the oocyte [49]. Small Rab GTPase family of proteins have been found to associate with Cgs and regulate their transport and exocytosis in oocytes of diverse organisms [43,44,50]. In mouse oocytes, for instance, positioning of Cgs to the oocyte cortex has been proposed to rely on both myosin Va motors localizing to these granules and inducing their movement on an intrinsically polarized bulk actin network, as well as “hitchhiking” on outward moving Rab11a-vesicles [43]. Our findings that Yg fusion and compaction to the oocyte center drive Cg movements towards the oocyte circumference, and that microtubule aster formation and translocation to the cortex lead to the concomitant accumulation of Rab11-positive vesicle at the circumference, identifies a yet unknown mechanism of Cg translocation and exocytosis during oocyte maturation. Why the mechanisms regulating Cg translocation and exocytosis differ between mice and zebrafish is still unknown, but likely the presence of Ygs in zebrafish but not mouse oocytes has led to different functional adaptations of cytoplasmic components.

Beyond the specific regulation of Cg localization within the maturing oocyte, our findings also shed light on the general mechanisms underlying cytoplasmic reorganization and the role of the cell cytoskeleton therein. Cytoskeletal networks, such as the actin and microtubule cytoskeleton, have been implicated as the main driving force underlying cytoplasmic organization in oocytes and embryos. In particular, myosin II–mediated actin network flows dragging the adjacent cytoplasm have been shown to generate cytoplasmic streaming in early *Drosophila* and zebrafish embryos [22,23], and the motion of kinesin I motors along microtubule arrays anchored to the cortex to drive cytoplasmic flows in *Drosophila* and *C*. *elegans* oocytes by exerting viscous drag forces to the surrounding ooplasm [18,20]. In addition, the assembly and interaction of actin comets and centrosomal microtubule asters with organelles such as Ygs, the nucleus, or the meiotic spindle can position those organelles to specific locations within oocytes and embryos [23–25]. Our findings that the partial disassembly of a prestressed microtubule network results in its transformation into acentrosomal microtubule asters, which move towards the oocyte cortex in a dynein-dependent manner, thereby carrying along and enriching Rab11-positive vesicles at the oocyte surface, suggest a novel mechanism by which the microtubule network can affect vesicle localization within cells. Moreover, our observation that such transformation of the microtubule network is driven by the cell cycle regulators Cdk1/CyclinB partially disassembling the microtubule network, mechanistically links cell cycle progression to cytoskeletal reorganization in cells.

One of the key findings of our study is that the regulated fusion and compaction of Ygs to the oocyte center can trigger outward cytoplasmic flows, which, in turn, transport Cgs towards the oocyte circumference. Importantly, the effect of Ygs fusion and compaction is independent of the role of bulk actomyosin flows in generating ooplasmic flows towards the oocyte AP, as in the PBb-treated oocytes, Yg fusion remains largely unchanged compared to control oocytes (S7I and S7I’ Fig). This suggests that beyond ATP/GTP hydrolysis-dependent actin/microtubule polymerization and the activity of their associated motor proteins, cytoplasmic flows and reorganization can also be achieved by coordinated changes in the size and shape of organelles. Interestingly, these are processes that—differently from the aforementioned cytoskeletal rearrangements—do not directly depend on the activity of motor proteins and polymerases but instead are regulated by changes in the propensity of organelles to deform and/or undergo fusion and compaction. Our observation that Yg fusion and compaction are mediated by a rise in the level of K^+^ within Ygs, which depends on the activity of Na^+^/K^+^ ATPase pumps and is triggered by GV breakdown, points at intracellular ion concentration as a critical regulator of organelle fusion and resultant cytoplasmic reorganization. Notably, an increase of intracellular K^+^ within the oocyte coinciding with the fusion of Ygs has previously been observed in oocytes of various teleosts, suggesting that K^+^-driven Yg fusion might represent an evolutionarily conserved mechanism in Yg-containing oocytes. How GV breakdown leads to an increase in K^+^ levels within Ygs and how such increase promotes Yg fusion is not yet understood, but it is conceivable that electrostatic interactions between the plasma membrane surrounding Ygs and K^+^ molecules promote the capacity of their plasma membrane to undergo fusion.

The different mechanisms identified in this study regulating blastodisc formation, and Cg translocation and decoration with Rab11, all depend on cell cycle progression and GV breakdown. In contrast, the molecular realization of these mechanisms does not seem to depend strictly on each other, as interference with one of these mechanisms does not necessarily lead to a complete failure in the progression of the remaining mechanisms. For instance, abrogating actomyosin flows does not affect microtubule aster formation and contraction or Yg fusion/compaction and, vice versa, interfering with the latter two processes does not prevent the release of actin into the ooplasm upon GV breakdown. However, these distinct mechanisms functionally interact in reorganizing ooplasmic components, with Yg fusion/compaction and actomyosin flow together triggering blastodisc formation, and Yg fusion/compaction and microtubules aster translocation together regulating exocytosis competency of Cgs and, thus, chorion elevation. These distinct molecular realizations of the contributing mechanisms, combined with their partially overlapping function, might not only confer robustness to the cytoplasmic reorganization, but also allow the independent use of these mechanisms in separate developmental processes.

Cytoplasmic reorganization relies on the inherent self-organizing properties of the cytoplasm and a combination of prepatterning of the cell and external signals modulating this self-organizing activity. While the external signals and prepatterning can vary depending on the specific organismal context, cytoplasmic self-organization represents a generic propensity of the cytoplasm that emerges as a result of a certain composition or state of the cytoplasm. Our finding of Yg fusion eliciting large-scale cytoplasmic streaming reveals a novel feature of such inherent self-organizing capacity of the cytoplasm that plays an important role in reorganizing the cytoplasm during zebrafish oocyte maturation. Given that Ygs constitute a major cytoplasmic component in oocytes from birds, reptiles, worms, and fish, Yg fusion might represent a common principle of regulating cytoplasmic streaming during oogenesis.

## Materials and methods

### Ethics statement

All animal procedures and protocols were performed in accordance with protocols (#66.018/0010-WF/II/3b/2014 incl. amendments 1–7) that were approved by the national Animal Experimentation Commission at the Federal Ministry of Austria in line with EU and national legislation, which includes an ethical evaluation of the performed experiments.

### Animal husbandry

Fish maintenance was carried out as described in [51]. In this study, 6- to 24-month-old females from WT AB, TL, and AB×TL strains as well as Tg(*hsp*:*Clip170-eGFP*), Tg(*actb1*:*Utr-GFP*), Tg(*Xla*.*Eef1a1*:*dclk2a-GFP*), Tg(*actb1*:*Utr-mCherry*);(*Xla*.*Eef1a1*:*dclk2a-GFP*), and Tg(*actb2*:*Rab11a-NeonGreen*) zebrafish lines were used.

### Ovarian follicle isolation

Methods of ovarian follicle isolation and culture were adapted from [5,52,53]. Female fish were anesthetized in 0.02% Tricaine and killed by decapitation. Ovaries were harvested in culture medium [90% Leibovitz’s L-15 medium with L-glutamine (Thermo Fisher) (pH 9.0), Penicillin–Streptomycin 50 U/ml, and 0.5% bovine serum albumin (BSA, Sigma-Aldrich)]. Follicles were isolated from ovaries by gentle pipetting with a glass Pasteur pipette and dissection with forceps. Stage III oocytes were identified by their size (500 to 690 μm), opaque cytoplasmic density, intact GV, and undetectable blastodisc [31] and kept in culture medium for up to 12 h at 25 to 26°C.

### Transgenic lines

For live imaging of F-actin and microtubules, oocytes from Tg*(actb1*:*Utr-GFP)* (actin), Tg*(Xla*.*Eef1a1*:*dclk2a-GFP*) (microtubules), and Tg*(actb1*:*Utr-mCherry*);(*Xla*.*Eef1a1*:*dclk2a-GFP*) were used [54,55]. Oocytes from Tg(*hsp*:*Clip170-eGFP*) were used for labeling of the ooplasm. To visualize subcellular Rab11 localization, the Tg*(actb2*:*Rab11a-NeonGreen)* zebrafish line ubiquitously expressing NeonGreen-tagged Rab11a (N-terminally tagged) was generated using the Tol2/Gateway technology [56,57]. Briefly, the coding sequence of RAB11a (NCBI reference sequence: NM_001007359.1) was amplified using gene-specific primers with additional Gateway recombination arms (5′ -GGGGACAGCTTTCTTGTACAAAGTGGCTATGGGGACACGAGACGACG −3′ and 5′- GGGGACAACTTTGTATAATAAAGTTGCCTAGATGCTCTGGCAGCACTGC -3′) from cDNA library of sphere stage wild-type Tübingen embryos. The PCR product was recombined with pDONR P2r-P3 (Lawson#211), and the resulting entry clone after sequence verification was recombined with pDestTol2pA2 (Chien#394), p5E b-actin promoter (Chien#229), and pME NeonGreen [58] with mNeonGreen licensed by Allele biotech to create pTol2-b-actin:NeonGreen-RAB11a. The pTol2 vector was coinjected with mRNA encoding the transposase (Invitrogen) into 1 cell-stage wild-type TL embryos. Individual positive carriers were selected and out-crossed with wild-type TL fish for stable single-copy genetic integration.

### mRNA injections

*PCS2-Rab11-mCherry* ([59]; N-terminally tagged) and *PCS2-Rab11S25N-mCherry* (see below; N-terminally tagged), *PCS2-DCLK-mKO2* (see below), *PCS2-Utrophin-GFP* [60], and *PCS2-CyclinB1-GFP* (Addgene plasmid #128426) expression constructs were used. mRNAs were synthesized using the SP6 mMessage mMachine Kit (Thermo Fisher). Injections into stage III oocytes were performed as described in [53] using glass capillary needles (30–0020, Harvard Apparatus, MA, USA), which were pulled by a needle puller (P-97, Sutter Instrument) and attached to a microinjection system (PV820, World Precision Instruments). The *PCS2-Rab11S25N-mCherry* plasmid was generated using Q5 Site-Directed Mutagenesis Kit (NEB) with *PCS2-Rab11-mCherry* plasmid and Rab11S25N-mCherry-Fwd (TGTGGGGAAGaatAACCTGCTGT) and Rab11S25N-mCherry-Rev (CCAGAGTCTCCAATTAGGACC) primers. The *PCS2-DCLK-mKO2* plasmid was constructed by amplifying the coding sequences of *DCLK1a-202-deltaK* (from *pT2KXIG-Xef1a-DCLK-GFP*, ZFIN ID: ZDB-TGCONSTRCT-090702-3) and *mKO2* (from *mKOkappa-2A-mTurquoise2*, Addgene plasmid # 98837) using gene-specific primers with overlapping arms DCLK1a-202_FOR (TGCAGGATCCCATATGGAGGAGCATTTTGACGA), DCLK1a-202_REV (taatcacactCCGATCTGAAATGGAGCTC), mKO2_FOR (TCAGATCGGagtgtgattaaaccagagatgaagatga), and mKO2_REV (TCACTATAGTTCTAGAGGCggaatgagctactgcatcttcta) and then cloned into ClaI-HF (NEB) and XhoI-HF (NEB) digested pCS2 plasmid (Lawson #444) with NEBuilder HiFi DNA Assembly Master Mix (NEB). The assembled plasmids were used for the transformation of chemically competent *E*. *coli* DH5α cells (NEB) and verified via Sanger sequencing. Approximately 350 pg *Rab11-mCherry*, 350 pg *Rab11S25N-mCherry*, 450 pg *DCLK-mKO2*, 200 pg *Utrophin-GFP*, and 300 pg *CyclinB1-GFP* mRNA were injected into random positions within stage III oocytes. The working concentrations of all mRNA solutions were diluted in 0.2M KCl as described [53]. For each experiment, between 100 and 200 oocytes were injected. The injected oocytes were incubated in culture medium (see Sample Preparation for Live Imaging section) for 3 to 4 h prior to maturation induction. Healthy oocytes, identified by their shape, cytoplasmic opacity, and intact GV, were sorted, and the remaining oocytes were removed from the medium. After 2 h, the culture medium was exchanged. Importantly, the humidity of the incubator in which the oocytes were cultured was ensured to be low, preventing oocytes from undergoing premature activation. The efficiency of injections varied from 10% to 30%.

### Sample preparation for live imaging

Oocytes were mounted in 0.5 ml 0.7% low melting point (LMP) agarose (Invitrogen) inside a glass bottom Petri dish (MatTek) and imaged on an inverted Leica SP5 or SP8 confocal microscope equipped with Leica 20× 0.7 NA, 20× 0.75 NA, or 40× 1.1 NA objectives or on a spinning disk setup (Andor Revolution Imaging System; Yokogawa CSU-X1) equipped with a Zeiss 40× 1.2 NA water immersion lens [54] for high-resolution imaging of microtubule asters. Images were rendered and processed using FIJI or Imaris (Bitplane). The temperature during imaging was kept constant at 26°C using a stage-heating device (Life Imaging Services). For drug treatment experiments, Colchicine (Sigma, 200 and 300 μM), Cycloheximide (Sigma, 700 μM), Cyto B (Sigma, 20 to 30 μg/ml), Dinaciclib (Selleckchem, 250 μM), DMSO (Sigma), PBb (Optopharma, 100 μM), and Taxol (Sigma, 12.5, 25, and 50 μM) were used. For labeling Ygs, oocytes were treated with Lysotracker Red (Invitrogen, 1 μM). Oocytes were kept in medium containing the drug (and/or Lysotracker) for 1 to 1.5 h and then transferred to drug-/Lysotracker-containing LMP agarose for 15 to 30 min. Prior to the start of imaging 6.5 ml culture medium with 1 μg/ml 17alpha,20beta-Dihydroxy-4-pregnen-3-one (DHP, diluted in 100% Ethanol, Sigma) containing drug/Lysotracker was added on top of the 0.5 ml solidified LMP agarose. To label the ooplasm or the GV nucleoplasm, 0.5 nl of 2 mg/ml of 10 KDa Dextran Alexa Fluor 647 (Invitrogen) diluted in 0.2 M KCl was injected into the oocyte center or the vicinity of GV, respectively.

### Blastodisc clearance analysis

Tg(*hsp*:*Clip170-eGFP*) oocytes were imaged during oocyte maturation using an inverted Leica SP5 or SP8 confocal microscope equipped with a Leica 20× objective. Oocytes with clear AV orientation were selected for further analysis. A kymograph was acquired along an 80 pix-wide line covering the oocyte AV axis. A line was then fitted to the blastodisc-Ygs interface in the kymograph over time until the end of oocyte maturation. Blastodisc clearance was measured from the tangent of the slope of the fitted line.

### Yolk granules fusion analysis

To determine the number of Ygs fusion events, the Lysotracker-exposed oocytes were imaged using an inverted Leica SP5 or SP8 confocal microscope equipped with a Leica 20× objective. Maximum intensity projection images of Ygs were then used to track the fusion dynamics of Ygs. Fusion events of 10 to 20 Ygs per oocyte were counted during oocyte maturation using Fiji. These results were then plotted as a histogram.

### Yolk granules, cortical granules, and ooplasm segmentation

To measure average Yg size over time, the Lysotracker-exposed oocytes were imaged using an inverted Leica SP5 or SP8 confocal microscope equipped with a Leica 20× objective. Maximum intensity projection images of Ygs were used to segment Yg (Lysotracker-signal positive) using Ilastik software. The segmented images were then analyzed in Fiji to measure the 2D cross-sectional area of Ygs over time.

For Cgs density at the oocyte surface, surface images (30 to 50 μm beneath the cortex) of Tg(*hsp*:*Clip170-eGFP*) or Tg(*actb2*:*Rab11a-NeonGreen*) oocytes exposed to Lysotracker were acquired using an inverted Leica SP5 or Sp8 confocal microscope equipped with a Leica 20× objective. Cgs were identified as ooplasm- as well as Lysotracker-negative granules and segmented using Ilastik software. The cross-sectional area of the imaging plane was also segmented with Ilastik, and the sum of the Cg area present on the imaging plane normalized to the area of the plane was measured as the Cg density at the oocyte surface over time using a custom-designed MATLAB script.

To measure the Cg distribution along the oocyte radius, a similar approach was employed on images taken at 50 to 75 μm beneath the cortex. Using the segmented images, oocyte center of mass and radius were determined, and the distance of each Cg to the oocyte center was measured and normalized to the oocyte radius. These results were then plotted as histogram with bin size of 0.05 along the normalized radial axis. Of note, the local decrease of CGs close to the cortex is the result of the segmentation procedure, not being able to precisely outline the position of the cortex relative to the small CGs present in this area. However, as this trend was observed in all of experimental conditions, it is unlikely to affect the interpretation of these experiments.

For performing phase fraction analysis, surface images of Tg(*hsp*:*Clip170-eGFP*) oocytes exposed to Lysotracker during 120 to 270 min after oocyte maturation induction were segmented using Ilastik software as described above to obtain Yg, Cg, ooplasm, and whole oocyte segmentations. Oocyte radius for angles between 0 and 360 degrees (with increments of 5 degrees) was then measured from oocyte segmentation. With the oocyte radius in hand, the distribution of binarized Yg, Cg, and ooplasm phases across all angles and over the oocyte radius was measured. These distributions were normalized to the total phase, the sum of all 3 phases, to obtain their relative phase distributions. The changes in relative phases of Yg, Cg, and ooplasm between 120 and 270 min were then calculated and plotted in the corresponding condition.

For Rab11 density at the surface of mature oocytes/eggs, Tg(*actb2*:*Rab11a-NeonGreen*) oocytes were incubated in 1 μg/ml DHP-containing medium for 270 min to complete maturation. Surface images (30 to 50 μm beneath the cortex) of the mature oocytes/eggs were acquired using an inverted Leica SP5 or Sp8 confocal microscope equipped with a Leica 20× objective. Rab11 vesicles were then segmented using Ilastik software. The cross-sectional area of the imaging plane was also segmented with Ilastik, and the sum of the Rab11 area present on the imaging plane, normalized to the area of the plane, was measured as the Rab11 density at the oocyte surface using a custom-designed MATLAB script.

### Yolk granules flow measurement

To measure the Yg flows, oocytes were exposed to Lysotracker for labeling Ygs and imaged with an inverted Leica SP8 confocal microscope equipped with a Leica 20×/40× objective. Multiple kymographs were acquired using Fiji along 10 pix-wide lines covering different oocyte axes. Yg flow speeds were then measured from the slopes of lines fitted to the outermost Ygs flowing towards the oocyte center in the kymographs.

### Oocyte volume measurement

To calculate changes in oocyte volume during oocyte maturation, Tg(*hsp*:*Clip170-eGFP*) oocytes were imaged in 3D with 125 Z slices, each 2 μm apart, covering 250 μm depth of the oocyte using an inverted Leica SP5 confocal microscope equipped with a Leica 10×/20× objective. The images were then segmented with Ilastik software to capture the complete oocyte shape, and the oocyte volume was measured as the sum of the number of pixels in segmented and binarized images and normalized to oocyte volume at the onset of the maturation process.

### Nucleoplasm distribution upon germinal vesicle breakdown

To measure nucleoplasm distribution upon GVBD, 10 KDa Dextran Alexa Fluor 647 was injected into or in the vicinity of the GV, resulting in the enrichment of Dextran in the GV. Next, a line scan was acquired using Fiji along a 30 pix-wide line covering the oocyte circumference, and Dextran intensity was measured along this line during the maturation process. The Dextran intensity profile was then subtracted from the profile prior to GVBD and normalized to its highest value.

### Chorion elevation assay

Stage III oocytes were injected with 350 pg of *Rab11-mCherry* or *Rab11S25N-mCherry* mRNA, incubated in culture medium for 3 h as described above, and then induced to undergo maturation by the addition of the DHP hormone for 270 min. In the last 30 min of oocyte maturation, the follicle membrane was removed manually using forceps [53]. The defolliculated fully mature oocytes/eggs were then transferred to a dish containing E3, triggering Cg exocytosis and, thereby, chorion elevation [28]. After 30 min in the E3 medium, the activated eggs were imaged with a Stereo-microscope, and the images were then used to calculate the extent of chorion elevation, measured as chorion diameter normalized to the oocyte diameter, using Fiji software [61].

### Phalloidin and immunohistochemistry

Stage III oocytes were harvested from wild-type female fish and induced to undergo maturation by the addition of DHP to the culture medium. After GVBD (1 to 1.5 h after maturation onset), the oocytes were fixed in a glass vial containing 2% paraformaldehyde at 4°C overnight. Fixed oocytes were then mounted in 4% LMP agarose and sectioned using a vibratome (VT1200S, Leica) into 200 μm thick slices. Sections were then washed 3× for 10 min in PBS with 0.1% Triton X-100, permeabilized by PBS with 0.5% Triton X-100 for 1 h at room temperature, and blocked in PBS containing 0.1% Triton X-100, 1% DMSO, and 10% goat serum (blocking buffer) for 3 h at room temperature. To visualize Dynein, Numa, or phosphorylated CyclinB levels/localization, the samples were incubated in anti-Dynein (clone 70.1, Sigma, dissolved 1:1,000 in blocking buffer), anti-Numa (Thermo Fisher, dissolved 1:100 in blocking buffer), and anti-Cyclin B1 (phospho S147, Abcam, dissolved 1:100 in blocking buffer) primary antibody at 4°C overnight, washed 3× for 10 min in PBS with 0.1% Triton X-100 at room temperature and incubated with secondary antibody (goat anti-mouse/rabbit conjugated to Alexa Fluor 488 (Molecular Probes), 1:250 in blocking buffer) for 3 h at room temperature. For visualizing the bulk actin gradient, the oocytes were fixed, sectioned, and permeabilized as described above, and the slices were then incubated in Phalloidin 488 (Invitrogen, dissolved 1:200 in blocking buffer) at 4°C overnight. The stained sections were imaged using an inverted Leica SP5 confocal microscope equipped with a Leica 20× objective.

### Bulk actin gradient analysis

Phalloidin-labelled and sectioned stage IV oocytes, which had just undergone GVBD, were imaged using an inverted Leica SP5 or Sp8 confocal microscope equipped with a Leica 20× objective. Oocytes with clear AV orientation were selected for further analysis. Maximum intensity projection images were used to segment bulk actin-containing ooplasmic pockets with Ilastik. The mean bulk actin intensity within these pockets was measured in Fiji and averaged over a 350-μm wide window centered along the oocyte AV axis.

### Oocyte extract preparation

To generate extracts of maturing oocytes, approximately 50 to 100 Tg(*actb1*:*Utr-GFP*) oocytes labelling F-actin were incubated in 3.5 ml L15 medium containing 1 μM Lysotracker and 1 μg/ml DHP for 60 to 90 min until GV broke down (oocytes were screened for GVBD under the stereomicroscope). Oocyte extracts were prepared by puncturing oocytes using a spike-micropipette with an inner diameter of 50 to 100 μm (BioMedical Instruments) attached to a syringe allowing for the aspiration of ooplasm-Ygs mixture. The pressure was manually controlled to prevent the aspiration of the medium into the pipette and thus the dilution of the oocyte extract. The ooplasm-Yg mixture was then promptly released into mineral oil (Sigma) inside a glass bottom Petri dish (MatTek) and imaged as described above 15 to 30 min after the extraction procedure had started.

### F-actin intensity measurement

To measure nuclear F-actin intensity within the GV shortly before and after GVBD in Tg(*actb1*:*Utr-GFP*) oocytes, 35 μm × 35 μm-shaped ROIs were defined within the GV, and the averaged intensities over time were obtained using Fiji. To measure F-actin intensity within the blastodisc of Tg(*actb1*:*Utr-GFP*) during oocyte maturation, a kymograph was acquired along a 50 pix-wide line covering the oocyte AV axis. A line scan was then performed at the blastodisc region to measure the F-actin intensity within the blastodisc region.

### Ooplasmic flow measurement

To measure ooplasmic flows, oocytes were injected with Dextran Alexa 647 to label ooplasm and imaged with an inverted Leica SP8 confocal microscope equipped with a Leica 20× objective. A kymograph was acquired using Fiji along a 50 pix-wide line covering the oocyte AV axis. Ooplasmic flow speeds were then measured from the slopes of lines fitted to the ooplasm pockets flowing towards the animal pole on the kymograph. The initial positions of those pockets were normalized to the length of the AV axis and used for obtaining the ooplasmic flow profile.

### CyclinB intensity measurement

To measure CyclinB intensity within the blastodisc of *CyclinB1-GFP* mRNA injected oocytes during oocyte maturation, a kymograph was acquired using Fiji along a 30 pix-wide line covering the oocyte circumference. A line scan was then performed at the blastodisc region to measure the CyclinB intensity there.

### Microtubule intensity measurement

To measure microtubule intensity within the blastodisc of Tg(*Xla*.*Eef1a1*:*dclk2a-GFP*) oocytes during oocyte maturation, a kymograph was acquired using Fiji along a 50 pix-wide line covering the oocyte AV axis. A line scan was then performed at the blastodisc region to measure the microtubule intensity there.

### Microtubule aster number and density

Microtubule asters were followed over time using Imaris 9.1.2 (using spot detection algorithm), and their tracking data were imported to MATLAB R2019b for obtaining their number over time. In cases where oocyte volume turned out to be different between different experimental conditions, microtubule aster density was calculated by normalizing the total aster number to the oocyte area.

### Microtubule cluster size analysis

The surface of Tg(*Xla*.*Eef1a1*:*dclk2a-GFP*) oocytes was imaged with an inverted Leica SP5 or SP8 confocal microscope equipped with a Leica 20× objective. Maximum intensity projections of these images (along the z slices) were used to obtain temporal intensity projections of microtubules during aster formation using the Temporal-Color Code plugin in Fiji. The temporally projected images were segmented with the Ilastik software to outline the microtubule structures undergoing contractions. Cluster sizes were then measured by applying the Voronoi algorithm plugin in Fiji to the segmented images. The size of the first and second biggest clusters was determined by a custom-designed MATLAB script.

### Microtubule flow measurement

To measure microtubule aster flow speed, Tg(*Xla*.*Eef1a1*:*dclk2a-GFP*) oocytes labeling microtubules were imaged with an inverted Leica SP8 confocal microscope equipped with a Leica 20×/40× objective. Multiple kymographs were acquired using Fiji along 10 pix-wide lines covering different oocyte axes. Microtubule aster flow speeds were then measured from the slopes of lines fitted to the microtubule asters flowing towards the oocyte surface in the kymographs.

### Rab11 flow measurement

To measure the speed of Rab11 vesicles, Tg(*actb2*:*Rab11a-NeonGreen*) oocytes (wild-type or exposed to 50 μM Taxol for 1 h) labeling Rab11 vesicles were imaged with an inverted Leica SP8 confocal microscope equipped with a Leica 40× objective. Multiple kymographs were acquired using Fiji along 10 pix-wide lines covering different oocyte axes. Rab11 flow speeds were then measured from the slopes of lines fitted to the Rab11 vesicles flowing towards the oocyte surface in the kymographs.

### Cortical granules depletion ratio analysis

To calculate the ratio of Cgs depletion upon egg activation, Tg*(actb2*:*Rab11a-NeonGreen)* oocytes labeling Rab11 were exposed to 1 μM Lysotracker marking Ygs and treated with DMSO (control), 200 μM Colchicine, 50 μM Taxol, or 100 μM Ouabain for 60 min. Consecutively, 1 μg/ml DHP was added to the solution initiating oocyte maturation, which was completed 270 min after the DHP addition. As described above, the culture medium was exchanged during the incubation process to improve oocyte quality and survival rate. Mature oocytes/eggs, identified by their cytoplasmic clarity (except for oocytes exposed to Ouabain) and their completion of GVBD, were mounted in 0.7% LMP agarose. Next, 6.5 ml E3 medium was added on top of the solidified agarose to initiate egg activation/Cg exocytosis, during which mature oocytes/eggs were imaged as detailed above. To measure the Cg depletion rate upon egg activation, Cgs in the last frame before and the first frame after the completion of egg activation were segmented using Ilastik software [62]. The depletion rate (in percentage) was then calculated as the ratio of Cgs that underwent exocytosis during activation and thus were absent in the final image compared to the stage before activation onset.

### Brightfield intensity measurement

To measure brightfield intensity during oocyte maturation, a 225 μm × 225 μm region was defined within the oocyte center, and the averaged intensities over time were obtained using Fiji.

### K^+^ vesicle number analysis

Superficial stacks of oocytes injected with 0.5 nl of 0.7 mg/ml ION Potassium Green-2 TMA+ Salt, K^+^ indicator (Abcam), and 2 mg/ml of 10 KDa Dextran Alexa Fluor 647 (to label ooplasm) diluted in nuclease-free water were imaged with an inverted Leica SP8 confocal microscope equipped with a Leica 40× objective. K^+^ vesicles were then segmented using Ilastik software, and their number was counted over time with a custom-designed MATLAB script.

### Quantification and statistical analysis

Statistical analysis was performed using Prism 8 (GraphPad Software). Data are represented as mean ± SEM and analyzed with the Mann–Whitney test. A *P* value < 0.05 was considered statistically significant. Sample size and *P* values are mentioned within the figure legends.

## Supporting information

S1 Fig Changes in oocyte volume and ooplasmic organization during oocyte maturation. (**A**) Left panels: Fluorescence images of stage III Tg(*hsp*:*clip170-GFP*) oocytes labeling ooplasm (magenta) before (stage III) and 120 and 270 min after maturation onset. Right panels: Segmentation views of the oocyte shown on the left. Orthogonal images (along the Z-axis) are shown in the bottom rows. (**A’**) Increase in oocyte volume during oocyte maturation normalized to its value at stage III (*N =* 3 experiments, *n =* 5 oocytes). See Table F in S1 Data for underlying data. (**B**) Fluorescence images of oocytes injected with Dextran Alexa 647 to mark GV nucleoplasm at the onset of, and 60 and 120 min post GVBD. Yellow lines along the oocyte circumference (Circum) and AV axis were used to obtain the kymographs in **B’**. (**B’**) Kymographs acquired along the circumference (left) and AV axis (right) of the oocyte shown on the left as a function of time. The white dashed lines mark the time point of GVBD. The black dashed line tracks the blastodisc interface. (**B”**) Changes in Dextran Alexa 647 signal injected to or in the vicinity of GV to label nucleoplasm, normalized to its distribution at the time point before GVBD, at 30 (cyan), 60 (magenta), and 120 min (green) after GVBD measured over the oocyte circumference (the yellow line in B, *N =* 1, *n =* 6). Circumferences of 0 and 800 μm correspond to the oocyte AP and VP, respectively. Asterisks track peak values for each curve. See Table G in S1 Data for underlying data. (**C**) Changes in phase fractions for ooplasm (left, green), Yg (middle, magenta), and Cg (right, cyan) between 120 and 270 min after imaging start in the absence of the maturation hormone DHP. Normalized (norm) radii of 0.5 and 1 correspond to the oocyte interior and cortex, respectively (*N =* 2, *n =* 6). See Table H in S1 Data for underlying data. (**D**) Schematic summarizing the ooplasmic reorganizations occurring during zebrafish oocyte maturation. At the onset of maturation, the GV (yellow) breaks down, triggering blastodisc formation at the AP of the oocyte. Concomitantly, Yg (magenta) fuse and compact towards the oocyte center, while Cg (cyan) translocate to the oocyte surface. Schematic in (**B**) demarcate the imaging plane used for obtaining the images in that panel. Error bars, SEM. AP, animal pole; AV, animal-vegetal; Cg, cortical granule; GV, germinal vesicle; GVBD, germinal vesicle breakdown; VP, vegetal pole; Yg, yolk granule.
(TIF)

S2 Fig The requirements of actin and microtubules cytoskeleton for blastodisc formation during oocyte maturation. (**A**) Fluorescence images of stage V Tg(*hsp*:*clip170-GFP*) oocytes labeling ooplasm (cyan) exposed to DMSO (control), 100 μM PBb (inhibiting myosin II activity), 30 μg/ml Cyto B (blocking actin polymerization), 700 μM CHX (blocking CyclinB synthesis, added at 45 min after maturation onset) or 200 μM Colchi (inhibiting microtubule polymerization). Yellow lines and arrowheads indicate the blastodisc height measured in (**A’**). (**A’**) Blastodisc clearance, measured as the height of blastodisc at the end of the maturation process as shown in (**A**), for oocytes exposed to DMSO (blue, *N =* 4 experiments, *n =* 29 oocytes), PBb (orange, *N* = 3, *n* = 19), Cyto B (magenta, *N* = 3, *n* = 15), CHX (red, *N* = 2, *n* = 18), or Colchi (dark green, *N* = 3, *n* = 14). See Table D in S2 Data for underlying data. (B) Brightfield images of oocytes exposed to DMSO, 250 μM Colchi, or 25 μM Taxol (stabilizing microtubules), which were induced to undergo oocyte maturation for 270 min and subsequently activated by exposure to E3 medium for 30 min. (B’) Chorion elevation, measured as chorion diameter normalized to the oocyte diameter, of oocytes exposed to DMSO (blue, control, *N =* 3, *n =* 44), Colchi (dark green, *N* = 3, *n* = 36), or Taxol (light green, *N* = 3, *n* = 34). See Table E in S2 Data for underlying data. Schematics in each panel demarcate the imaging plane used for obtaining the images in that panel. Error bars, SEM. AP, animal pole; Cg, cortical granule; CHX, Cycloheximide; Colchi, Colchicine; Cyto B, Cytochalasin B; PBb, para-Nitroblebbistatin; VP, vegetal pole; Yg, yolk granule.
(TIF)

S3 Fig Oocyte staging and CyclinB subcellular localization during oocyte maturation. (**A**) Fluorescence images of stage III Tg(*actb1*:*Utr-mCherry*);(*Xla*.*Eef1a1*:*dclk2a-GFP*) oocytes labeling F-actin (gray, top row) and microtubules (purple, bottom row) before (stage III) and 150, 170, and 180 min after maturation onset. The dashed box indicates the region used for acquiring the kymographs in (**A’**). The solid box (i) indicates the region used for the zoomed-in view shown in the insets. White arrowheads in the insets mark meiotic spindle formation at the end of the first meiosis. “High” and “Low” refer to the intensity of F-actin in the ooplasm. (**A’**) Kymographs of F-actin (top) and microtubules (bottom) acquired along the AV axis of the oocyte in (**A**) as a function of time. The yellow and green arrowheads mark the appearance and disappearance of F-actin in the bulk of the ooplasm, respectively. The white arrowheads mark the first meiotic spindle. Meiosis stages are indicated according to spindle formation and positioning within the oocyte. (**B**) Bulk actin intensity normalized to its value at GVBD onset and measured at the AP, the 35 μm × 35 μm white box shown in Fig 2A, of Tg(*actb1*:*Utr-GFP*) oocytes over time (*N =* 2 experiments, *n =* 9 oocytes). The dashed lines in (**B**) and (**C**) denote the time point of GVBD. See Table F in S2 Data for underlying data. (**C**) CyclinB intensity normalized to its value at GVBD onset and measured at the AP, first 400–500 μm along the circumference line shown in Fig 2F and 2F’, of oocytes injected with *CyclinB-GFP* mRNA over time (*N =* 2, *n =* 7). Meiosis stages in (**B**) and (**C**) are indicated according to CyclinB dynamics. See Table G in S2 Data for underlying data. (**D**) Brightfield (top) and fluorescence (bottom) images of stage IV oocytes sectioned and stained with anti-pCyclinB antibody before and after GVBD. The dashed lines indicate the GV region. Schematics in each panel demarcate the imaging plane used for obtaining the images in that panel. Error bars, SEM. AP, animal pole; AV, animal-vegetal; GV, germinal vesicle; GVBD, germinal vesicle breakdown; VP, vegetal pole.
(TIF)

S4 Fig Microtubule reorganization and Dynein/Numa subcellular localization during oocyte maturation. (**A**) Kymographs acquired along the AV axis of oocytes exposed to 250 μM Dinaciclib as a function of time. Green arrowhead marks the time point of oocyte exposure to Dinaciclib, and white arrowheads mark the transformation of the microtubule asters to a more homogeneous distribution of microtubules. (**B**) Microtubule aster density for oocytes exposed to DMSO (blue, *N =* 2 experiments, *n =* 8 oocytes) or 20 μg/ml Cyto B (magenta, *N* = 2, *n* = 6) during oocyte maturation. See Table F in S3 Data for underlying data. (**C**) Microtubule contraction type distribution (1 for normal asters, 0.5 for large-scale contractions, and 0 for abnormal asters) in Tg(*Xla*.*Eef1a1*:*dclk2a-GFP*) oocytes labelling microtubules exposed to DMSO (blue, *N* = 2, *n* = 9) or Cili D (red, *N* = 2, *n* = 37). See Table G in S3 Data for underlying data. (**D**) Top row: Temporal maximum projection of Tg(*Xla*.*Eef1a1*:*dclk2a-GFP*) oocytes exposed to DMSO together with DHP (left column), 75 μM of Cili D together with DHP (middle column) and 280 μM Colchi without DHP (immature oocyte, right column) from the onset to the end of contractions. Middle row: Voronoi triangulation of the microtubule networks shown in the upper row, delimited by magenta lines. Bottom row: Overlay of the microtubule networks and their corresponding Voronoi triangulation. (**D’**) Size of the first and second biggest microtubule clusters for oocytes exposed to DMSO together with DHP (blue, *N* = 2, *n* = 9), 75 μM Cili D together with DHP (magenta, *N* = 2, *n* = 19) or 280 μM Colchi without DHP (green, immature oocytes, *N* = 2, *n* = 8) obtained from images in (**D**). The black dashed line demarcates the position where the sizes of the first and second clusters are equal. See Table H in S3 Data for underlying data. (**E**) Zoomed-in fluorescence images of stage III Tg(*Xla*.*Eef1a1*:*dclk2a-GFP*) injected with Dextran Alexa 647 next to the GV (black dashed line) for marking nucleoplasm before and after GVBD. Top row, microtubules; bottom row, Dextran. The white asterisk demarcates microtubule depolymerization upon GVBD at the animal pole. (**F**) Left: Immunofluorescence image of a stage IV oocyte sectioned and stained with anti-Dynein antibody. The yellow box (i) indicates the region used for the zoom-in view on the right. Right: Zoomed-in view of the image on the left at the indicated ROI (i). Arrowheads point at the cortical localization of Dynein. (**F’**) Left: Immunofluorescence image of a stage IV oocyte sectioned and stained with anti-Numa antibody. The yellow box (ii) indicates the region used for the inset zoom-in view on the right. Right: Zoomed-in view of the image on the left at the indicated ROI (ii). Arrowheads point at the cortical localization of Numa. (**G**) Fluorescence images of stage III Tg(*Xla*.*Eef1a1*:*dclk2a-GFP*) oocytes labeling microtubules exposed to DMSO (top row) or 75 μM Cili D (bottom row) before (stage III) and 120 and 150 min after maturation onset. Green and white arrowheads demarcate microtubule asters below the oocyte surface (central portion of the imaging plane) and at the oocyte surface (marginal region of the imaging plane), respectively. The dashed boxes indicate the lateral regions used for acquiring the kymographs in (**G’**). (**G’**) Kymographs acquired along the lateral axis of oocytes exposed to DMSO (top) or Cili D (bottom) as a function of time. Green and white arrowheads demarcate microtubule asters below the oocyte surface and at the oocyte surface, respectively. Schematics in each panel demarcate the imaging plane used for obtaining the images in that panel. Error bars, SEM. AP, animal pole; AV, animal-vegetal; Cili D, Ciliobrevin D; Colchi, Colchicine; Cyto B, Cytochalasin B; GV, germinal vesicle; GVBD, germinal vesicle breakdown; VP, vegetal pole.
(TIF)

S5 Fig Cg translocation during oocyte maturation. (**A**) Top row: Fluorescence images of stage III Tg(*hsp*:*clip170-GFP*) oocytes labelling ooplasm (cyan) exposed to Lysotracker to mark Ygs (magenta) 120 and 270 min after maturation induction for oocytes treated with DMSO, 200 μM Colchi, 100 μM PBb, or 100 μM Ouabain. Cgs (black) were identified by their exclusion of both Clip-170-GFP and Lysotracker. Bottom row: Segmented Cg obtained from the images in the top row. White dashed lines mark the oocyte outline. (**A’**) Cg density profile along the oocyte radius at 120 and 270 min after maturation onset for oocytes exposed to DMSO (green, *N =* 3 experiments, *n =* 11 oocytes), Colchi (blue, *N* = 3, *n* = 15), PBb (orange, *N =* 2, *n =* 12), or Ouabain (red, *N* = 3, *n* = 14). Normalized (norm.) radius of 0 and 1 correspond to the oocyte center and surface, respectively. Note that changes, but not the absolute values, in Cg distribution along the oocyte radius between 120 and 270 min reflect Cg translocation/movement during this time. See Table D in S4 Data for underlying data. Schematic in panel (A) demarcates the imaging plane used for obtaining the images in that panel. Error bars, SEM. Cg, cortical granule; Colchi, Colchicine; PBb, para-Nitroblebbistatin; Yg, yolk granule.
(TIF)

S6 Fig Rab11 subcellular localization during oocyte maturation. (**A**) Fluorescence images of stage-V Tg(*actb2*:*Rab11a-NeonGreen*) oocytes labelling Rab11^+^ vesicles. Left, WT oocytes; right, oocytes exposed to 200 μM Colchi. Images on the right of the fluorescence images show segmented Rab11^+^ vesicles obtained from the images on the left. (**A’**) Density of Rab11-positive vesicles, measured from the images in (**A**) for WT (blue, *N =* 2 experiments, *n =* 15 oocytes) and Colchi-treated oocytes (green, *N* = 2, *n* = 15). See Table E in S4 Data for underlying data. (**B**) Fluorescence images of Tg(*actb2*:*Rab11a-NeonGreen*) oocytes marking Rab11-positive vesicles (green) and exposed to Lysotracker to label Ygs (magenta) pre- (left), during (middle), and post-activation (right) with E3 medium and treated with DMSO (control, top), 200 μM Colchicine (Colchi, middle), or 50 μM Taxol (bottom). Cgs (black) are identified by their exclusion of Lysotracker and Rab11 signal. On the right of the fluorescence images (for pre- and post-activation) segmented Cg are shown. On the right of the fluorescence images (for activation) zoomed-in images are shown of the ROI demarcated by the white dashed boxes in the fluorescence images. Arrowheads mark exemplary Cg undergoing exocytosis. (**B’**) Cg depletion ratio upon egg activation for oocytes exposed to DMSO (blue, *N =* 3, *n =* 12), 200 μM Colchi (green, *N* = 3, *n* = 14) or 50 μM Taxol (black, *N* = 3, *n* = 10). See Table F in S4 Data for underlying data. (**C**) Microtubule aster density (count in 10^5^ μm^2^) for oocytes injected with 350 pg *Rab11-mcherry* (blue, *N* = 3, *n* = 9) or *Rab11S25N-mCherry* (magenta; dominant negative, *N =* 3, *n =* 9) mRNA during oocyte maturation. See Table G in S4 Data for underlying data. (**D**) Changes in phase fractions of Yg (magenta) and Cg (cyan) for oocytes injected with 350 pg *Rab11-mcherry* (left, *N* = 2, *n* = 6) or *Rab11S25N-mCherry* (right, *N* = 2, *n* = 6) between 120 and 270 min after maturation onset. Normalized (norm) radii of 0.5 and 1 correspond to the oocyte interior and cortex, respectively. See Table H in S4 Data for underlying data. Schematics in each panel demarcate the imaging plane used for obtaining the images in that panel. Error bars, SEM. Mann–Whitney test, **p = 0.0012. Cg, cortical granule; Colchi, Colchicine;WT, wild-type; Yg, yolk granule.
(TIF)

S7 Fig Subcellular K^+^ localization and Yg fusion during oocyte maturation. (**A**) Brightfield intensity of oocytes exposed to DMSO (control, blue, *N =* 3 experiments, *n =* 15 oocytes) or 100 μM Ouabain (magenta, *N* = 3, *n* = 14) within a 225 μm × 225 μm region in the oocyte center normalized to its value at stage III during oocyte maturation. See Table D in S5 Data for underlying data. (**B**) Temporal projection of segmented Ygs in oocytes exposed to DMSO (control, left) or Ouabain (right) (same oocytes as in Fig 5B) between 120 and 270 min after maturation onset. (**C**) Fluorescence images of stage V Tg(*hsp*:*clip170-GFP*) oocytes labeling the ooplasm exposed to DMSO (control, left) or 100 μM Ouabain (right). Ygs and Cgs are depicted by their exclusion of ooplasmic signal. Blue lines mark the blastodisc height as measured in (**C’**). (**C’**) Blastodisc clearance, measured as the height of blastodisc at the end of the maturation process, for oocytes exposed to DMSO (control, blue, *N* = 4, *n* = 29) or 100 μM Ouabain (magenta, *N* = 3, *n* = 13). See Table E in S5 Data for underlying data. (**D**) Maximum fluorescence intensity projection of oocytes exposed to Lysotracker to mark Yg 120 min after maturation onset. (**D’**) Yg area averaged for the first, second, and third hour (h) after maturation onset along the oocyte AV axis (*N =* 3, *n =* 8). See Table F in S5 Data for underlying data. (**E**) Fluorescence images of stage III Tg(*actb1*:*Utr-GFP*) oocytes labeling F-actin exposed to 100 μM Ouabain before (stage III) and 60, 120, and 270 min after maturation onset. The dashed line indicates the GV region. (**F**) Fluorescence images of stage III Tg(*Xla*.*Eef1a1*:*dclk2a-GFP*) oocytes labeling microtubules exposed to DMSO (top) or 100 μM Ouabain (bottom) at 60, 120, 180, 240, and 270 min after maturation onset. (**F’**) Microtubule aster density for oocytes exposed to DMSO (blue, *N =* 3, *n =* 9) or Ouabain (magenta, *N* = 2, *n* = 7) during oocyte maturation. See Table G in S5 Data for underlying data. (**G**) Fluorescence images of Tg(*actb2*:*Rab11a-NeonGreen*) oocytes marking Rab11-positive vesicles (green) and exposed to Lysotracker to label Yg (magenta) pre- (left) and during (right) activation with E3 medium for oocytes exposed to DMSO (control, top) or 100 μM Ouabain (bottom). Cg are identified by their exclusion of Lysotracker and the ooplasmic signal. The images on the right of the fluorescence images (for pre-activation) show segmented Cg. The images on the right of the fluorescence images (for activation) are zoomed-in images of the regions outlined by the white dashed boxes in the fluorescence images. Arrowheads mark the colocalization of Rab11-positive vesicles with Cg. (**H**) Fluorescence images (top row) of stage III oocytes injected with K^+^ indicator (K^+^-Green, green) and Dextran Alexa Fluor 647 to mark ooplasm (magenta) exposed to 100 μM Ouabain before (stage III) and 150 and 270 min after maturation onset. Cg (black) are identified by their exclusion of both Dextran and K^+^-Green. Images in bottom row show segmented K^+^ vesicles identified from the images in the top row. Dashed lines mark the oocyte outline. (**H’**) Number of K^+^ vesicles in superficial stacks of WT oocytes (blue, *N* = 2, *n* = 8, same data as in Fig 5C’) or oocytes exposed to 100 μM Ouabain (magenta, *N* = 2, *n* = 12) during maturation. See Table H in S5 Data for underlying data. (**I**) Fluorescence images of stage III Tg(*hsp*:*clip170-GFP*) oocytes labelling the ooplasm (cyan) exposed to Lysotracker to mark Yg (magenta) for oocytes and treated with 100 μM PBb before (stage III) and 120, 180, and 270 min after maturation onset. Cg (black) were identified by their exclusion of both Clip-170-GFP and Lysotracker. (**I’**) Increase in average Yg area at the end of oocyte maturation normalized to its value at stage III for oocytes exposed to DMSO (green, *N* = 3, *n* = 15) or PBb (orange, *N* = 3, *n* = 7). See Table I in S5 Data for underlying data. Schematics in each panel demarcate the imaging plane used for obtaining the images in that panel. Error bars, SEM. Mann–Whitney test, ns (C’): *p* = 0.0679, ns (I’): *p* = 0.2101. AP, animal pole; AV, animal-vegetal; Cg, cortical granule; GV, germinal vesicle; PBb, para-Nitroblebbistatin; VP, vegetal pole; WT, wild-type; Yg, yolk granule.
(TIF)

S1 Data Data underlying Figs 1A”, 1B’, 1C’, 1D’, 1D”’, S1A’, S1B” and S1C. (XLSX)

S2 Data Data underlying Figs 2B’, 2D’, 2F’, S2A’, S2B’, S3B, and S3C. (XLSX)

S3 Data Data underlying Figs 3A”, 3B’, 3C’, 3E, 3F’, S4B, S4C, and S4D’. (XLSX)

S4 Data Data underlying Figs 4A”, 4C’, 4D’, S5A’, S6A’, S6B’, S6C, and S6D. (XLSX)

S5 Data Data underlying Figs 5A’, 5B’, 5C’, S7A, S7C’, S7D’, S7F’, S7H’, and S7I’. (XLSX)

S1 Movie Germinal vesicle breakdown triggers blastodisc formation. Related to Fig 1. Time-lapse brightfield (left) and fluorescence (right) movies (42-s interval) of an exemplary Tg(*hsp*:*clip170-GFP*) oocyte labeling the ooplasm during oocyte maturation. Time: 0 to 280 min after maturation induction. Scale bar: 100 μm.
(AVI)

S2 Movie Yolk granules undergo fusion upon germinal vesicle breakdown. Related to Fig 1. Time-lapse fluorescence movie (42-s interval) of maximum intensity projection of an exemplary wild-type oocyte exposed to Lysotracker to label yolk granules during oocyte maturation. Time: 0 to 155 min after maturation induction. Scale bar: 100 μm.
(AVI)

S3 Movie Yolk granules compact towards the oocyte center, and cortical granules move to the oocyte cortex during oocyte maturation. Related to Fig 1. Time-lapse fluorescence movie (123-s interval) of an exemplary Tg(*hsp*:*clip170-GFP*) oocyte marking the ooplasm (cyan) and exposed to Lysotracker labeling yolk granules (magenta) during oocyte maturation. Time: 120 to 270 min after maturation induction. Scale bar: 100 μm.
(AVI)

S4 Movie Intact actomyosin and cell cycle progression are required for blastodisc formation during oocyte maturation. Related to S2 Fig. Time-lapse fluorescence movie of exemplary Tg(*hsp*:*clip170-GFP*) oocytes labeling the ooplasm and exposed to DMSO (control, 225-s interval), 100 μM para-Nitroblebbistatin (PBb, 280-s interval), 30 μg/ml Cytochalasin B (Cyto B, 304-s interval), 700 μM Cycloheximide (CHX, 386-s interval, added at 45 min after maturation onset) and 200 μM Colchicine (Colchi, 265-s interval) during oocyte maturation. Time: 0 to 270 min after maturation induction. Scale bars: 100 μm.
(AVI)

S5 Movie The actin cytoskeleton rearranges during oocyte maturation. Related to Fig 2. Time-lapse fluorescence movie (182-s interval) of an exemplary Tg(*actb1*:*Utr-GFP*) oocyte labeling F-actin during oocyte maturation. Time: 0 to 270 min after maturation induction. Scale bar: 100 μm.
(AVI)

S6 Movie Actin flows in the oocyte extract are directed towards the extract center. Related to Fig 2. Time-lapse fluorescence movie (67-s interval) of an exemplary extract generated from Tg(*actb1*:*Utr-GFP*) oocytes labeling F-actin (gray) and exposed to Lysotracker labeling yolk granules (magenta) during oocyte maturation. Time: 30 to 90 min after maturation induction. Scale bar: 50 μm.
(AVI)

S7 Movie Ooplasmic flows towards the oocyte animal pole are short range. Related to Fig 2. Time-lapse fluorescence movie (223-s interval) of an exemplary wild-type oocyte injected with Dextran Alexa 647 to mark the ooplasm. Time: 0 to 270 min after maturation induction. Scale bar: 100 μm.
(AVI)

S8 Movie Bulk actin network reorganization coincides with cell cycle progression. Related to S3 Fig. Time-lapse fluorescence movies (54-s interval) of an exemplary Tg(*actb1*:*Utr-mCherry*);(*Xla*.*Eef1a1*:*dclk2a-GFP*) oocyte labeling F-actin (yellow, left movie) and microtubules (purple, right movie), respectively, during oocyte maturation. Time: 0 to 270 min after maturation induction. Scale bar: 100 μm.
(AVI)

S9 Movie CyclinB dynamics during oocyte maturation. Related to Fig 2. Time-lapse fluorescence movie (138-s interval) of an exemplary wild-type oocyte injected with *CyclinB-GFP* mRNA to visualize CyclinB dynamics. Time: 0 to 270 min after maturation induction. Scale bar: 100 μm.
(AVI)

S10 Movie Microtubule network forms asters upon germinal vesicle breakdown. Related to Fig 3. Time-lapse fluorescence movie (29-s interval) of an exemplary Tg(*Xla*.*Eef1a1*:*dclk2a-GFP*) oocyte labeling microtubules during oocyte maturation. Time: 0 to 270 min after maturation induction. Scale bar: 100 μm.
(AVI)

S11 Movie Microtubule network forms asters upon germinal vesicle breakdown. Related to Fig 3. Maximum intensity projection high-resolution time-lapse fluorescence movie (52-s interval) of an exemplary Tg(*Xla*.*Eef1a1*:*dclk2a-GFP*) oocyte marking microtubules during oocyte maturation. Time: 0 to 270 min after maturation induction. Scale bar: 50 μm.
(AVI)

S12 Movie Microtubule network transformation is cell cycle dependent. Related to Fig 3. Time-lapse fluorescence movies of exemplary Tg(*Xla*.*Eef1a1*:*dclk2a-GFP*) oocytes labeling microtubules and exposed to DMSO (left movie, 159-s interval, added at 95 min), 700 μM Cycloheximide (CHX, middle movie, 204-s interval, added at 85 min), or 250 μM Dinaciclib (right movie, 165-s interval, added at 38 min) during oocyte maturation. Time: 0 to 270 min after maturation induction. Scale bars: 100 μm.
(AVI)

S13 Movie Microtubule aster formation relies on dynein activity. Related to Fig 3. Time-lapse fluorescence movies of exemplary Tg(*Xla*.*Eef1a1*:*dclk2a-GFP*) oocytes labeling microtubules and exposed to 75 μM Ciliobrevin D (Cili D, 225-s interval, left movie: large-scale microtubule contraction, right movie: abnormal aster formation), during oocyte maturation. Time: 0 to 200 min after maturation onset. Scale bar: 100 μm.
(AVI)

S14 Movie Partial microtubule depolymerization drives microtubule aster formation during oocyte maturation. Related to Fig 3. Time-lapse fluorescence movies of exemplary immature Tg(*Xla*.*Eef1a1*:*dclk2a-GFP*) oocytes (in the absence of DHP) labeling microtubules exposed to DMSO (control, left movie, 161-s interval, added at 72 min) or 300 μM Colchicine (Colchi, right movie, 144-s interval, added at 67 min). Time: 0 to 456 min after incubation. Scale bar: 100 μm.
(AVI)

S15 Movie Microtubule asters flow towards the oocyte cortex during oocyte maturation. Related to Fig 4. Time-lapse fluorescence movie (30-s interval) of an exemplary Tg(*Xla*.*Eef1a1*:*dclk2a-GFP*) oocyte labeling microtubules during oocyte maturation. Time: 120 to 270 min after maturation induction. Scale bar: 100 μm.
(AVI)

S16 Movie Rab11 decorates the surface of cortical granules upon egg activation. Related to Fig 4. Time-lapse fluorescence movie (72-s interval) of an exemplary mature Tg(*actb2*:*Rab11a-NeonGreen*) oocyte/egg marking Rab11-positive vesicles (green) and exposed to Lysotracker to label yolk granules (magenta) during egg activation. Time: 0 to 144 min after exposure to E3 inducing activation. Scale bar: 50 μm.
(AVI)

S17 Movie Rab11 vesicles colocalize and flow together with microtubule asters towards oocyte cortex. Related to Fig 4. Time-lapse fluorescence movies (90-s interval) of an exemplary Tg(*actb2*:*Rab11a-NeonGreen*) oocyte marking Rab11-positive vesicles (magenta, left movie) and injected with *DCLK-mKO2* mRNA to label microtubules (green, middle) during oocyte maturation. Right movie is overlay of left (Rab11) and middle (microtubules) movies. Time: 120 to 270 min after maturation induction. Scale bar: 100 μm.
(AVI)

S18 Movie Rab11 vesicles colocalize and flow together with microtubule asters towards the cortex of Taxol-treated oocytes. Related to Fig 4. Time-lapse fluorescence movies (72-s interval) of an exemplary Tg(*actb2*:*Rab11a-NeonGreen*) oocyte marking Rab11-positive vesicles (magenta, left movie) and injected with *DCLK-mKO2* mRNA to label microtubules (green, middle movie) and exposed to 50 μM Taxol during oocyte maturation. Right movie is overlay of left (Rab11) and middle (microtubules) movies. Time: 120 to 270 min after maturation induction. Scale bar: 100 μm.
(AVI)

S19 Movie Yolk granule fusion is required for cortical granule translocation towards the oocyte cortex. Related to S5 Fig. Time-lapse fluorescence movies of exemplary Tg(*hsp*:*clip170-GFP*) oocytes labeling the ooplasm (cyan) and exposed to Lysotracker, marking yolk granules (magenta), and DMSO (control), 200 μM Colchicine (Colchi), 100 μM para-Nitroblebbistatin (PBb), or 100 μM Ouabain (30-min interval) during oocyte maturation. Time: 0 to 270 min after maturation induction. Scale bars: 100 μm.
(AVI)

S20 Movie Yolk granule fusion relies on Na^+^/K^+^ ATPase activity. Related to Fig 5. Time-lapse fluorescence movies of exemplary wild-type zebrafish oocytes exposed to Lysotracker, labeling yolk granules, DMSO (control, left movie, 164-s interval), or 100 μM Ouabain (right movie, 108-s interval) during oocyte maturation. Time: 0 to 270 min after maturation induction. Scale bars: 100 μm.
(AVI)

S21 Movie Yolk granule compaction to the center and cortical granule translocation towards the oocyte cortex depend on Na^+^/K^+^ ATPase activity. Related to Fig 5. Time-lapse fluorescence movies of exemplary Tg(*hsp*:*clip170-GFP*) oocytes labeling the ooplasm (cyan) and exposed to Lysotracker, marking yolk granules (magenta), and DMSO (control, 145-s interval, left movie) or 100 μM Ouabain (right movie, 167-s interval) during oocyte maturation. Time: 120 to 270 min after maturation induction. Scale bar: 100 μm.
(AVI)

S22 Movie K^+^ concentration increase upon germinal vesicle breakdown triggers yolk granule fusion. Related to Fig 5. Time-lapse fluorescence movies of exemplary oocytes injected with Dextran Alexa 647 to mark the ooplasm and K^+^ indicator (K^+^ green) during oocyte maturation. Left movie: wild-type oocytes (control, 232-s interval). Right movie: oocytes exposed to 100 μM Ouabain (172-s interval). Time: 0 to 270 min after maturation induction. Scale bars: 50 μm.
(AVI)

## Abbreviations

AV: animal-vegetal
Cg: cortical granule
Cyto B: Cytochalasin B
GV: germinal vesicle
GVBD: germinal vesicle breakdown
LMP: low melting point
PBb: para-Nitroblebbistatin
Yg: yolk granule

## References

[1] Wallace RA, Selman K. Cellular and Dynamic Aspects of Oocyte Growth in Teleosts. Am Zool. 1981 May;21(2):325–343.

[2] Almonacid M, Ahmed WW, Bussonnier M, Mailly P, Betz T, Voituriez R, et al. Active diffusion positions the nucleus in mouse oocytes. Nat Cell Biol. 2015 Apr;17(4):470–479. doi: 10.1038/ncb3131 25774831

[3] Azoury J, Lee KW, Georget V, Rassinier P, Leader B, Verlhac MH. Spindle positioning in mouse oocytes relies on a dynamic meshwork of actin filaments. Curr Biol. 2008 Oct;18(19):1514–1519. doi: 10.1016/j.cub.2008.08.044 18848445

[4] Campbell PD, Heim AE, Smith MZ, Marlow FL. Kinesin-1 interacts with Bucky ball to form germ cells and is required to pattern the zebrafish body axis. Development. 2015 Sep;142(17):2996–3008. doi: 10.1242/dev.124586 26253407 PMC4582183

[5] Elkouby YM, Jamieson-Lucy A, Mullins MC. Oocyte Polarization Is Coupled to the Chromosomal Bouquet, a Conserved Polarized Nuclear Configuration in Meiosis. Brickman JM, editor. PLoS Biol. 2016 Jan;14(1):e1002335. 26741740 10.1371/journal.pbio.1002335 PMC4704784

[6] Lénárt P, Bacher CP, Daigle N, Hand AR, Eils R, Terasaki M, et al. A contractile nuclear actin network drives chromosome congression in oocytes. Nature. 2005 Aug;436(7052):812–818. doi: 10.1038/nature03810 16015286

[7] Marlow FL, Mullins MC. Bucky ball functions in Balbiani body assembly and animal–vegetal polarity in the oocyte and follicle cell layer in zebrafish. Dev Biol. 2008 Sep;321(1):40–50. doi: 10.1016/j.ydbio.2008.05.557 18582455 PMC2606906

[8] Prodon F, Chenevert J, Sardet C. Establishment of animal-vegetal polarity during maturation in ascidian oocytes. Dev Biol. 2006 Feb;290(2):297–311. doi: 10.1016/j.ydbio.2005.11.025 16405883

[9] Prodon F, Sardet C, Nishida H. Cortical and cytoplasmic flows driven by actin microfilaments polarize the cortical ER-mRNA domain along the a-v axis in ascidian oocytes. Dev Biol. 2008 Jan;313(2):682–699. doi: 10.1016/j.ydbio.2007.11.001 18062956

[10] Quinlan ME. Cytoplasmic Streaming in the Drosophila Oocyte. Annu Rev Cell Dev Biol. 2016;32:173–195. doi: 10.1146/annurev-cellbio-111315-125416 27362645

[11] Schuh M, Ellenberg J. A new model for asymmetric spindle positioning in mouse oocytes. Curr Biol. 2008 Dec;18(24):1986–1992. doi: 10.1016/j.cub.2008.11.022 19062278

[12] Yi K, Rubinstein B, Unruh JR, Guo F, Slaughter BD, Li R. Sequential actin-based pushing forces drive meiosis I chromosome migration and symmetry breaking in oocytes. J Cell Biol. 2013 Mar;200(5):567–576. doi: 10.1083/jcb.201211068 23439682 PMC3587830

[13] Mitchison TJ, Field CM. Self-Organization of Cellular Units. Annu Rev Cell Dev Biol. 2021 Oct 6;37(1):23–41. doi: 10.1146/annurev-cellbio-120319-025356 34186005 PMC9059766

[14] Shamipour S, Caballero-Mancebo S, Heisenberg CP. Cytoplasm’s Got Moves. Dev Cell. 2021 Jan;56(2):213–226. 33321104 10.1016/j.devcel.2020.12.002

[15] Cheng X, Ferrell JE. Spontaneous emergence of cell-like organization in *Xenopus* egg extracts. Science. 2019 Nov;366(6465):631–637. 31672897 10.1126/science.aav7793 PMC7839252

[16] Ierushalmi N, Malik-Garbi M, Manhart A, Abu Shah E, Goode BL, Mogilner A, et al. Centering and symmetry breaking in confined contracting actomyosin networks. Elife. 2020 Apr;9. doi: 10.7554/eLife.55368 32314730 PMC7173961

[17] Sakamoto R, Tanabe M, Hiraiwa T, Suzuki K, Ishiwata S, Maeda YT, et al. Tug-of-war between actomyosin-driven antagonistic forces determines the positioning symmetry in cell-sized confinement. Nat Commun. 2020;11(1):3063. doi: 10.1038/s41467-020-16677-9 32541780 PMC7295813

[18] Kimura K, Mamane A, Sasaki T, Sato K, Takagi J, Niwayama R, et al. Endoplasmic-reticulum-mediated microtubule alignment governs cytoplasmic streaming. Nat Cell Biol. 2017;19(4):399–406. doi: 10.1038/ncb3490 28288129

[19] Meaders JL, de Matos SN, Burgess DR. A Pushing Mechanism for Microtubule Aster Positioning in a Large Cell Type. Cell Rep. 2020 Oct;33(1):108213. doi: 10.1016/j.celrep.2020.108213 33027648

[20] Monteith CE, Brunner ME, Djagaeva I, Bielecki AM, Deutsch JM, Saxton WM. A Mechanism for Cytoplasmic Streaming: Kinesin-Driven Alignment of Microtubules and Fast Fluid Flows. Biophys J. 2016;110(9):2053–2065. doi: 10.1016/j.bpj.2016.03.036 27166813 PMC4939475

[21] Xie J, Minc N. Cytoskeleton Force Exertion in Bulk Cytoplasm. Front Cell Dev Biol. 2020;8:69. doi: 10.3389/fcell.2020.00069 32117991 PMC7031414

[22] Deneke VE, Puliafito A, Krueger D, Narla AV, De Simone A, Primo L, et al. Self-Organized Nuclear Positioning Synchronizes the Cell Cycle in Drosophila Embryos. Cell. 2019;177(4):925–941.e17. doi: 10.1016/j.cell.2019.03.007 30982601 PMC6499673

[23] Shamipour S, Kardos R, Xue SL, Hof B, Hannezo E, Heisenberg CP. Bulk Actin Dynamics Drive Phase Segregation in Zebrafish Oocytes. Cell. 2019;177(6):1463–1479.e18. doi: 10.1016/j.cell.2019.04.030 31080065

[24] Li H, Guo F, Rubinstein B, Li R. Actin-driven chromosomal motility leads to symmetry breaking in mammalian meiotic oocytes. Nat Cell Biol. 2008 Nov;10(11):1301–1308. doi: 10.1038/ncb1788 18836438

[25] Theriot JA, Mitchison TJ, Tilney LG, Portnoy DA. The rate of actin-based motility of intracellular Listeria monocytogenes equals the rate of actin polymerization. Nature. 1992 May;357(6375):257–260. doi: 10.1038/357257a0 1589024

[26] Selman K, Wallace RA, Sarka A, Qi X. Stages of oocyte development in the zebrafish, Brachydanio rerio. J Morphol. 1993 Nov;218(2):203–224. doi: 10.1002/jmor.1052180209 29865471

[27] Bontems F, Stein A, Marlow F, Lyautey J, Gupta T, Mullins MC, et al. Bucky Ball Organizes Germ Plasm Assembly in Zebrafish. Curr Biol. 2009 Mar;19(5):414–422. doi: 10.1016/j.cub.2009.01.038 19249209

[28] Kanagaraj P, Gautier-Stein A, Riedel D, Schomburg C, Cerdà J, Vollack N, et al. Souffle/Spastizin Controls Secretory Vesicle Maturation during Zebrafish Oogenesis. Ketting RF, editor. PLoS Genet. 2014 Jun;10(6):e1004449. doi: 10.1371/journal.pgen.1004449 24967841 PMC4072560

[29] Fernández J, Valladares M, Fuentes R, Ubilla A. Reorganization of cytoplasm in the zebrafish oocyte and egg during early steps of ooplasmic segregation. Dev Dyn. 2006 Mar;235(3):656–671. doi: 10.1002/dvdy.20682 16425221

[30] Fuentes R, Mullins MC, Fernández J. Formation and dynamics of cytoplasmic domains and their genetic regulation during the zebrafish oocyte-to-embryo transition. Mech Dev. 2018;154:259–269. doi: 10.1016/j.mod.2018.08.001 30077623

[31] Lessman CA. Oocyte maturation: Converting the zebrafish oocyte to the fertilizable egg. Gen Comp Endocrinol. 2009 Mar;161(1):53–57. doi: 10.1016/j.ygcen.2008.11.004 19027744

[32] Becker KA, Hart NH. Reorganization of filamentous actin and myosin-II in zebrafish eggs correlates temporally and spatially with cortical granule exocytosis. J Cell Sci. 1999 Jan 1;112(1):97–110. doi: 10.1242/jcs.112.1.97 9841907

[33] Bischof J, Brand CA, Somogyi K, Májer I, Thome S, Mori M, et al. A cdk1 gradient guides surface contraction waves in oocytes. Nat Commun. 2017;8(1):849. doi: 10.1038/s41467-017-00979-6 29021609 PMC5636809

[34] Belmont LD, Hyman AA, Sawin KE, Mitchison TJ. Real-time visualization of cell cycle-dependent changes in microtubule dynamics in cytoplasmic extracts. Cell. 1990 Aug;62(3):579–589. doi: 10.1016/0092-8674(90)90022-7 2379239

[35] Ishihara K, Nguyen PA, Wühr M, Groen AC, Field CM, Mitchison TJ. Organization of early frog embryos by chemical waves emanating from centrosomes. Philos Trans R Soc B. 2014 Sep;369(1650):20130454. doi: 10.1098/rstb.2013.0454 25047608 PMC4113098

[36] Verde F. Taxol-induced microtubule asters in mitotic extracts of Xenopus eggs: requirement for phosphorylated factors and cytoplasmic dynein. J Cell Biol. 1991 Mar;112(6):1177–1187. doi: 10.1083/jcb.112.6.1177 1671864 PMC2288889

[37] Verde F, Dogterom M, Stelzer E, Karsenti E, Leibler S. Control of microtubule dynamics and length by cyclin A- and cyclin B-dependent kinases in Xenopus egg extracts. J Cell Biol. 1992 Sep;118(5):1097–1108. doi: 10.1083/jcb.118.5.1097 1387400 PMC2289588

[38] Foster PJ, Fürthauer S, Shelley MJ, Needleman DJ. Active contraction of microtubule networks. eLife. 2015 Dec;4:e10837. doi: 10.7554/eLife.10837 26701905 PMC4764591

[39] Alvarado J, Sheinman M, Sharma A, MacKintosh FC, Koenderink GH. Molecular motors robustly drive active gels to a critically connected state. Nat Phys. 2013 Sep;9(9):591–597.

[40] Roostalu J, Rickman J, Thomas C, Nédélec F, Surrey T. Determinants of Polar versus Nematic Organization in Networks of Dynamic Microtubules and Mitotic Motors. Cell. 2018;175(3):796–808.e14. doi: 10.1016/j.cell.2018.09.029 30340043 PMC6198040

[41] Okumura M, Natsume T, Kanemaki MT, Kiyomitsu T. Dynein–Dynactin–NuMA clusters generate cortical spindle-pulling forces as a multi-arm ensemble. Elife. 2018 May;7:e36559. doi: 10.7554/eLife.36559 29848445 PMC6037482

[42] Kotak S, Busso C, Gönczy P. Cortical dynein is critical for proper spindle positioning in human cells. J Cell Biol. 2012 Oct;199(1):97–110. doi: 10.1083/jcb.201203166 23027904 PMC3461507

[43] Cheeseman LP, Boulanger J, Bond LM, Schuh M. Two pathways regulate cortical granule translocation to prevent polyspermy in mouse oocytes. Nat Commun. 2016 Dec;7(1):13726. doi: 10.1038/ncomms13726 27991490 PMC5187413

[44] Sato M, Grant BD, Harada A, Sato K. Rab11 is required for synchronous secretion of chondroitin proteoglycans after fertilization in *Caenorhabditis* elegans. J Cell Sci. 2008 Oct;121(19):3177–3186. 18765566 10.1242/jcs.034678

[45] Zhen Y, Stenmark H. Cellular functions of Rab GTPases at a glance. J Cell Sci. 2015 Jan 1:jcs.166074. doi: 10.1242/jcs.166074 26272922

[46] Scheffler K, Uraji J, Jentoft I, Cavazza T, Mönnich E, Mogessie B, et al. Two mechanisms drive pronuclear migration in mouse zygotes. Nat Commun. 2021 Dec;12(1):841. doi: 10.1038/s41467-021-21020-x 33547291 PMC7864974

[47] Chan CJ, Costanzo M, Ruiz-Herrero T, Mönke G, Petrie RJ, Bergert M, et al. Hydraulic control of mammalian embryo size and cell fate. Nature. 2019 Jul;571(7763):112–116. doi: 10.1038/s41586-019-1309-x 31189957

[48] Selman K, Wallace RA, Cerdà J. Bafilomycin A1 inhibits proteolytic cleavage and hydration but not yolk crystal disassembly or meiosis during maturation of sea bass oocytes: Inhibition of Oocyte Hydration by Bafilomycin A1. J Exp Zool. 2001 Aug;290(3):265–278. 11479906 10.1002/jez.1057

[49] Liu M. The biology and dynamics of mammalian cortical granules. Reprod Biol Endocrinol. 2011;9(1):149. doi: 10.1186/1477-7827-9-149 22088197 PMC3228701

[50] Rojas J, Hinostroza F, Vergara S, Pinto-Borguero I, Aguilera F, Fuentes R, et al. Knockin’ on Egg’s Door: Maternal Control of Egg Activation That Influences Cortical Granule Exocytosis in Animal Species. Front Cell Dev Biol. 2021 Sep 3;9:704867. doi: 10.3389/fcell.2021.704867 34540828 PMC8446563

[51] Westerfield M. THE ZEBRAFISH BOOK A guide for the laboratory use of zebrafish Danio (Brachydanio) rerio. 4th ed. University of Oregon Press; 2000.

[52] Elkouby YM, Mullins MC. Methods for the analysis of early oogenesis in Zebrafish. Dev Biol. 2017 Oct;430(2):310–324. doi: 10.1016/j.ydbio.2016.12.014 27988227 PMC5555829

[53] Nair S, Lindeman RE, Pelegri F. In vitro oocyte culture-based manipulation of zebrafish maternal genes. Dev Dyn. 2013 Jan;242(1):44–52. doi: 10.1002/dvdy.23894 23074011 PMC3857710

[54] Behrndt M, Salbreux G, Campinho P, Hauschild R, Oswald F, Roensch J, et al. Forces Driving Epithelial Spreading in Zebrafish Gastrulation. Science. 2012 Oct;338(6104):257–260. doi: 10.1126/science.1224143 23066079

[55] York AG, Parekh SH, Nogare DD, Fischer RS, Temprine K, Mione M, et al. Resolution doubling in live, multicellular organisms via multifocal structured illumination microscopy. Nat Methods. 2012 Jul;9(7):749–754. doi: 10.1038/nmeth.2025 22581372 PMC3462167

[56] Kwan KM, Fujimoto E, Grabher C, Mangum BD, Hardy ME, Campbell DS, et al. The Tol2kit: A multisite gateway-based construction kit forTol2 transposon transgenesis constructs. Dev Dyn. 2007 Nov;236(11):3088–3099. doi: 10.1002/dvdy.21343 17937395

[57] Villefranc JA, Amigo J, Lawson ND. Gateway compatible vectors for analysis of gene function in the zebrafish. Dev Dyn. 2007 Nov;236(11):3077–3087. doi: 10.1002/dvdy.21354 17948311 PMC4518551

[58] Shaner NC, Lambert GG, Chammas A, Ni Y, Cranfill PJ, Baird MA, et al. A bright monomeric green fluorescent protein derived from Branchiostoma lanceolatum. Nat Methods. 2013 May;10(5):407–409. doi: 10.1038/nmeth.2413 23524392 PMC3811051

[59] Nowak M, Machate A, Yu SR, Gupta M, Brand M. Interpretation of the FGF8 morphogen gradient is regulated by endocytic trafficking. Nat Cell Biol. 2011 Feb;13(2):153–158. doi: 10.1038/ncb2155 21258372

[60] Burkel BM, von Dassow G, Bement WM. Versatile fluorescent probes for actin filaments based on the actin-binding domain of utrophin. Cell Motil Cytoskeleton. 2007 Nov;64(11):822–832. doi: 10.1002/cm.20226 17685442 PMC4364136

[61] Schindelin J, Arganda-Carreras I, Frise E, Kaynig V, Longair M, Pietzsch T, et al. Fiji: an open-source platform for biological-image analysis. Nat Methods. 2012 Jul;9(7):676–682. doi: 10.1038/nmeth.2019 22743772 PMC3855844

[62] Sommer C, Straehle C, Kothe U, Hamprecht FA. Ilastik: Interactive learning and segmentation toolkit. 2011 IEEE International Symposium on Biomedical Imaging: From Nano to Macro [Internet]. Chicago, IL, USA: IEEE; 2011 [cited 2022 Feb 13]. p. 230–233. Available from: http://ieeexplore.ieee.org/document/5872394/

