# Supplementary figures and images for: Yolk granule fusion and microtubule aster formation regulate cortical granule translocation and exocytosis in zebrafish oocytes
Source: PLoS Biol. 2023 Jun 8;21(6):e3002146. doi: 10.1371/journal.pbio.3002146 (PMC10284390; doi:10.1371/journal.pbio.3002146)

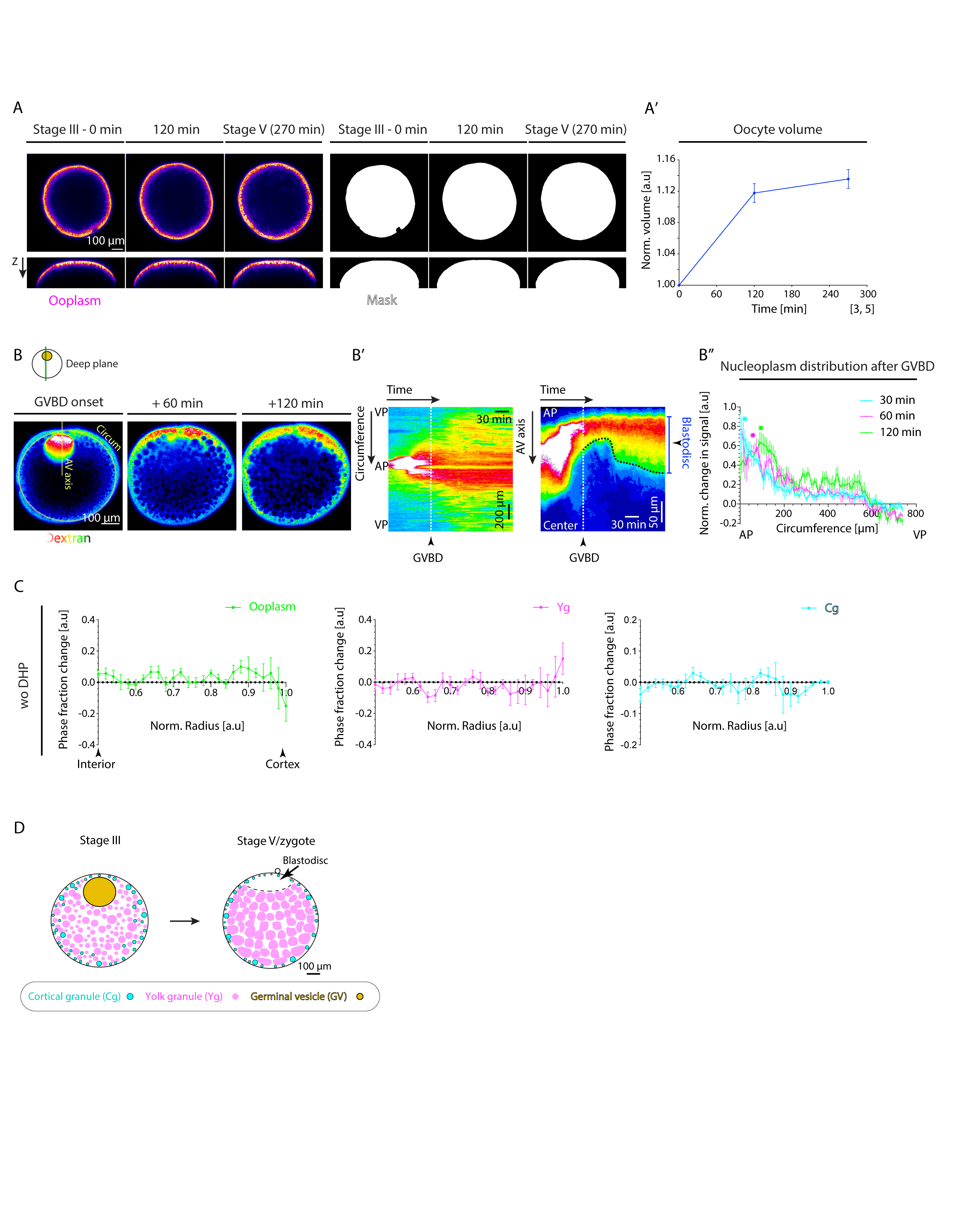

Supplement: S1 Fig — (A) Left panels: Fluorescence images of stage III Tg(hsp:clip170-GFP) oocytes labeling ooplasm (magenta) before (stage III) and 120 and 270 min after maturation onset. Right panels: Segmentation views of the oocyte shown on the left. Orthogonal images (along the Z-axis) are shown in the bottom rows. (A’) Increase in oocyte volume during oocyte maturation normalized to its value at stage III (N = 3 experiments, n = 5 oocytes). See Table F in S1 Data for underlying data. (B) Fluorescence images of oocytes injected with Dextran Alexa 647 to mark GV nucleoplasm at the onset of, and 60 and 120 min post GVBD. Yellow lines along the oocyte circumference (Circum) and AV axis were used to obtain the kymographs in B’. (B’) Kymographs acquired along the circumference (left) and AV axis (right) of the oocyte shown on the left as a function of time. The white dashed lines mark the time point of GVBD. The black dashed line tracks the blastodisc interface. (B”) Changes in Dextran Alexa 647 signal injected to or in the vicinity of GV to label nucleoplasm, normalized to its distribution at the time point before GVBD, at 30 (cyan), 60 (magenta), and 120 min (green) after GVBD measured over the oocyte circumference (the yellow line in B, N = 1, n = 6). Circumferences of 0 and 800 μm correspond to the oocyte AP and VP, respectively. Asterisks track peak values for each curve. See Table G in S1 Data for underlying data. (C) Changes in phase fractions for ooplasm (left, green), Yg (middle, magenta), and Cg (right, cyan) between 120 and 270 min after imaging start in the absence of the maturation hormone DHP. Normalized (norm) radii of 0.5 and 1 correspond to the oocyte interior and cortex, respectively (N = 2, n = 6). See Table H in S1 Data for underlying data. (D) Schematic summarizing the ooplasmic reorganizations occurring during zebrafish oocyte maturation. At the onset of maturation, the GV (yellow) breaks down, triggering blastodisc formation at the AP of the oocyte. Concomitantly, [file pbio.3002146.s001.tif]

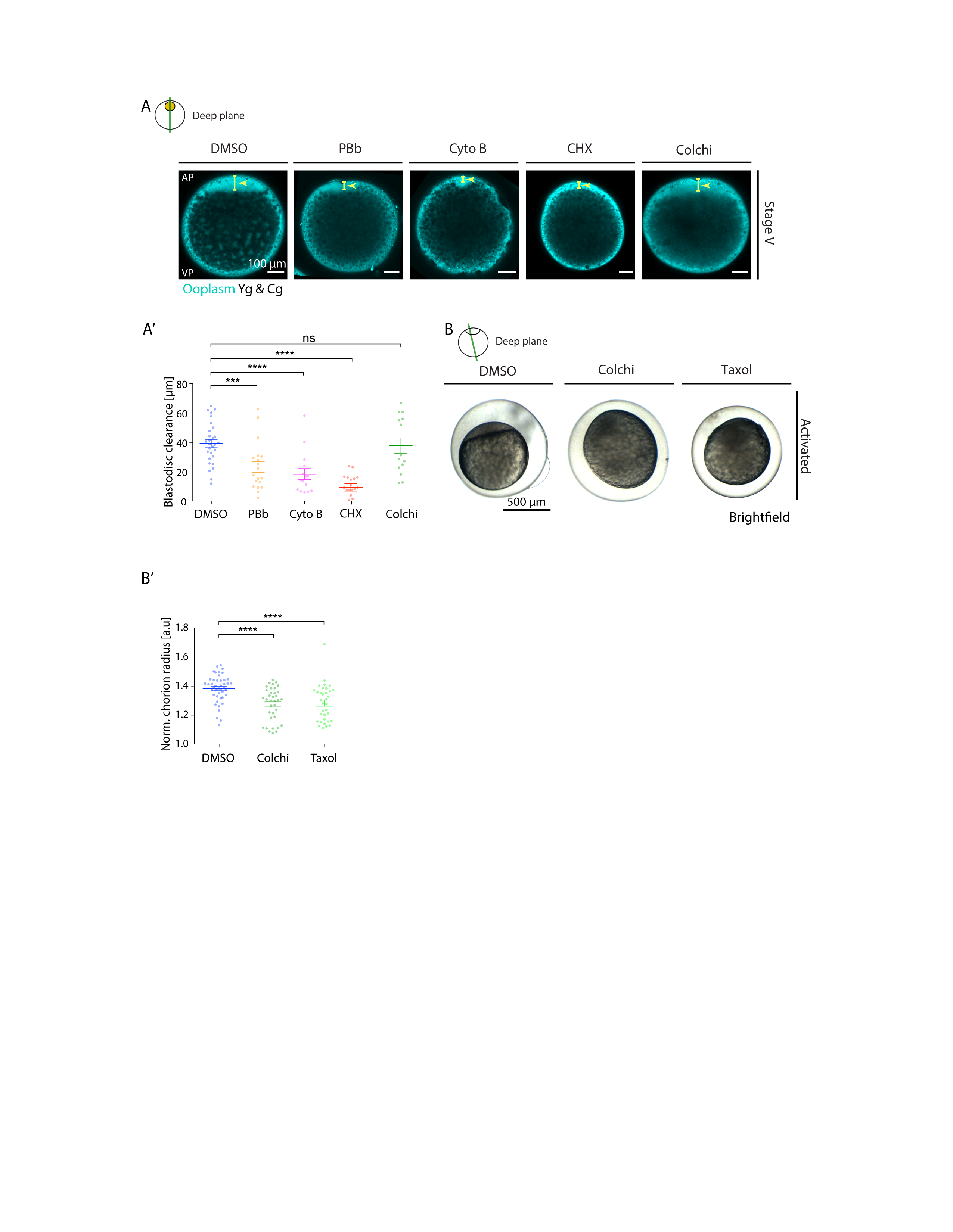

Supplement: S2 Fig — (A) Fluorescence images of stage V Tg(hsp:clip170-GFP) oocytes labeling ooplasm (cyan) exposed to DMSO (control), 100 μM PBb (inhibiting myosin II activity), 30 μg/ml Cyto B (blocking actin polymerization), 700 μM CHX (blocking CyclinB synthesis, added at 45 min after maturation onset) or 200 μM Colchi (inhibiting microtubule polymerization). Yellow lines and arrowheads indicate the blastodisc height measured in (A’). (A’) Blastodisc clearance, measured as the height of blastodisc at the end of the maturation process as shown in (A), for oocytes exposed to DMSO (blue, N = 4 experiments, n = 29 oocytes), PBb (orange, N = 3, n = 19), Cyto B (magenta, N = 3, n = 15), CHX (red, N = 2, n = 18), or Colchi (dark green, N = 3, n = 14). See Table D in S2 Data for underlying data. (B) Brightfield images of oocytes exposed to DMSO, 250 μM Colchi, or 25 μM Taxol (stabilizing microtubules), which were induced to undergo oocyte maturation for 270 min and subsequently activated by exposure to E3 medium for 30 min. (B’) Chorion elevation, measured as chorion diameter normalized to the oocyte diameter, of oocytes exposed to DMSO (blue, control, N = 3, n = 44), Colchi (dark green, N = 3, n = 36), or Taxol (light green, N = 3, n = 34). See Table E in S2 Data for underlying data. Schematics in each panel demarcate the imaging plane used for obtaining the images in that panel. Error bars, SEM. AP, animal pole; Cg, cortical granule; CHX, Cycloheximide; Colchi, Colchicine; Cyto B, Cytochalasin B; PBb, para-Nitroblebbistatin; VP, vegetal pole; Yg, yolk granule. (TIF) [file pbio.3002146.s002.tif]

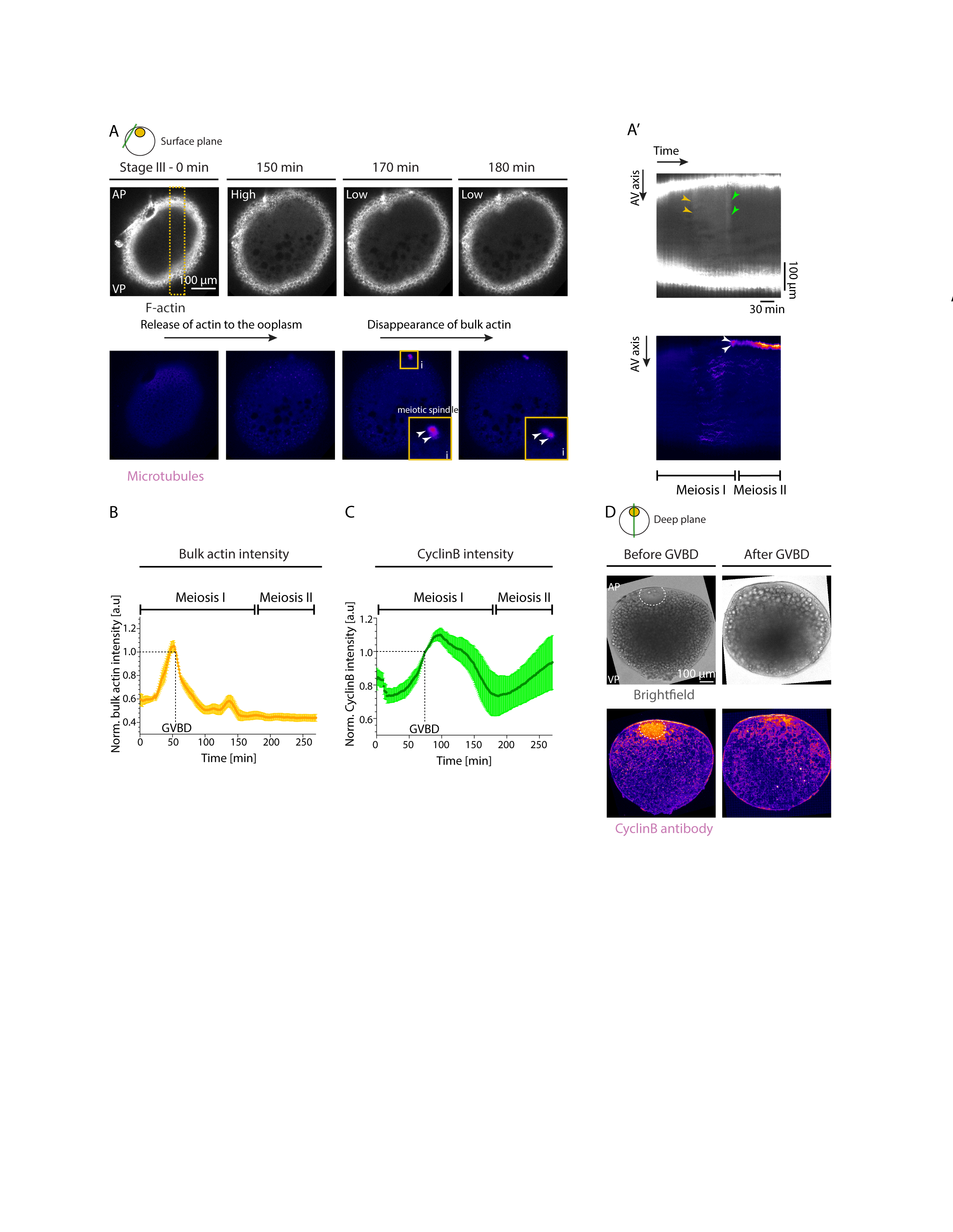

Supplement: S3 Fig — (A) Fluorescence images of stage III Tg(actb1:Utr-mCherry);(Xla.Eef1a1:dclk2a-GFP) oocytes labeling F-actin (gray, top row) and microtubules (purple, bottom row) before (stage III) and 150, 170, and 180 min after maturation onset. The dashed box indicates the region used for acquiring the kymographs in (A’). The solid box (i) indicates the region used for the zoomed-in view shown in the insets. White arrowheads in the insets mark meiotic spindle formation at the end of the first meiosis. “High” and “Low” refer to the intensity of F-actin in the ooplasm. (A’) Kymographs of F-actin (top) and microtubules (bottom) acquired along the AV axis of the oocyte in (A) as a function of time. The yellow and green arrowheads mark the appearance and disappearance of F-actin in the bulk of the ooplasm, respectively. The white arrowheads mark the first meiotic spindle. Meiosis stages are indicated according to spindle formation and positioning within the oocyte. (B) Bulk actin intensity normalized to its value at GVBD onset and measured at the AP, the 35 μm × 35 μm white box shown in Fig 2A, of Tg(actb1:Utr-GFP) oocytes over time (N = 2 experiments, n = 9 oocytes). The dashed lines in (B) and (C) denote the time point of GVBD. See Table F in S2 Data for underlying data. (C) CyclinB intensity normalized to its value at GVBD onset and measured at the AP, first 400–500 μm along the circumference line shown in Fig 2F and 2F’, of oocytes injected with CyclinB-GFP mRNA over time (N = 2, n = 7). Meiosis stages in (B) and (C) are indicated according to CyclinB dynamics. See Table G in S2 Data for underlying data. (D) Brightfield (top) and fluorescence (bottom) images of stage IV oocytes sectioned and stained with anti-pCyclinB antibody before and after GVBD. The dashed lines indicate the GV region. Schematics in each panel demarcate the imaging plane used for obtaining the images in that panel. Error bars, SEM. AP, animal pole; AV, animal-vegetal; GV, germinal vesicle; GVBD, germinal vesic [file pbio.3002146.s003.tif]

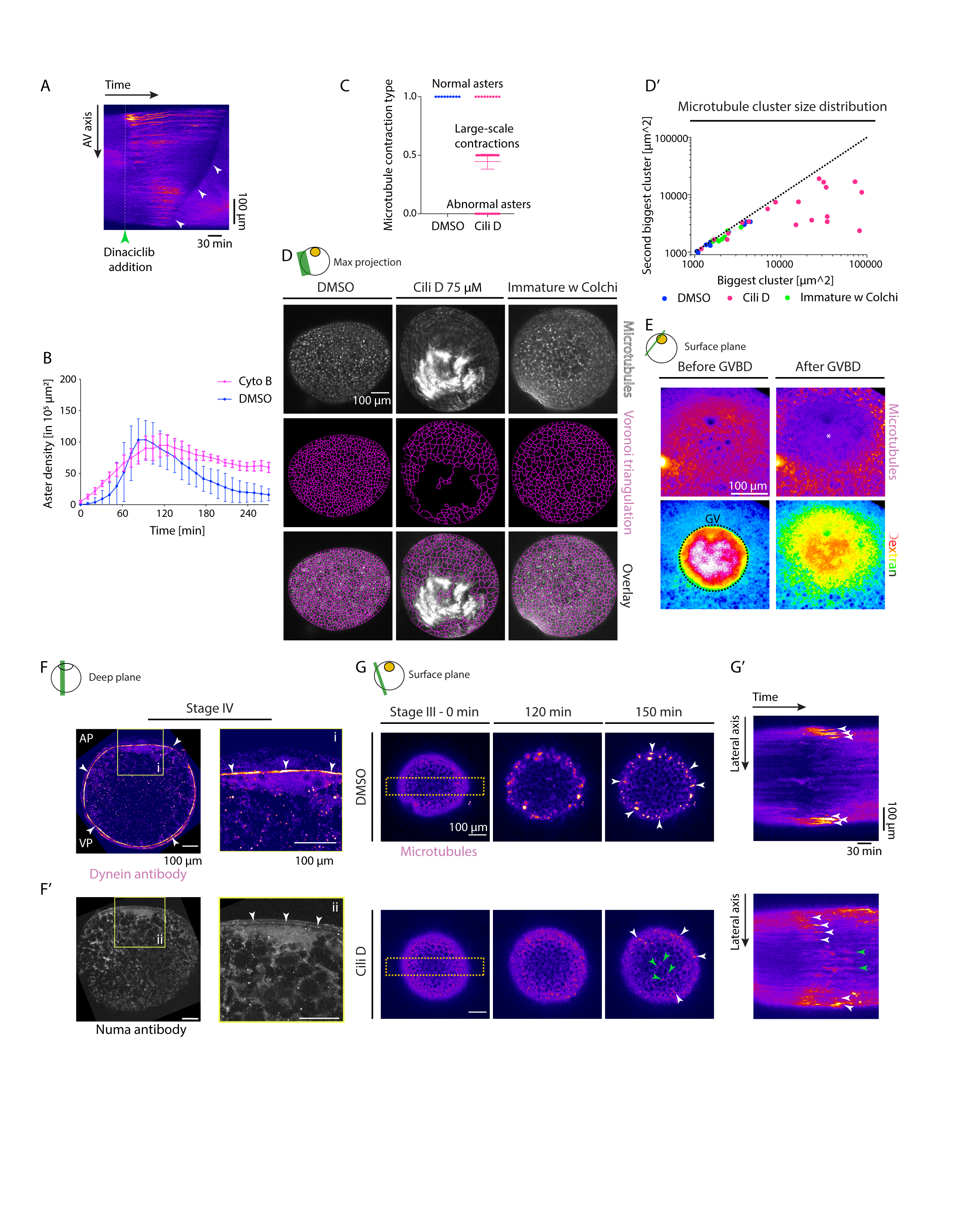

Supplement: S4 Fig — (A) Kymographs acquired along the AV axis of oocytes exposed to 250 μM Dinaciclib as a function of time. Green arrowhead marks the time point of oocyte exposure to Dinaciclib, and white arrowheads mark the transformation of the microtubule asters to a more homogeneous distribution of microtubules. (B) Microtubule aster density for oocytes exposed to DMSO (blue, N = 2 experiments, n = 8 oocytes) or 20 μg/ml Cyto B (magenta, N = 2, n = 6) during oocyte maturation. See Table F in S3 Data for underlying data. (C) Microtubule contraction type distribution (1 for normal asters, 0.5 for large-scale contractions, and 0 for abnormal asters) in Tg(Xla.Eef1a1:dclk2a-GFP) oocytes labelling microtubules exposed to DMSO (blue, N = 2, n = 9) or Cili D (red, N = 2, n = 37). See Table G in S3 Data for underlying data. (D) Top row: Temporal maximum projection of Tg(Xla.Eef1a1:dclk2a-GFP) oocytes exposed to DMSO together with DHP (left column), 75 μM of Cili D together with DHP (middle column) and 280 μM Colchi without DHP (immature oocyte, right column) from the onset to the end of contractions. Middle row: Voronoi triangulation of the microtubule networks shown in the upper row, delimited by magenta lines. Bottom row: Overlay of the microtubule networks and their corresponding Voronoi triangulation. (D’) Size of the first and second biggest microtubule clusters for oocytes exposed to DMSO together with DHP (blue, N = 2, n = 9), 75 μM Cili D together with DHP (magenta, N = 2, n = 19) or 280 μM Colchi without DHP (green, immature oocytes, N = 2, n = 8) obtained from images in (D). The black dashed line demarcates the position where the sizes of the first and second clusters are equal. See Table H in S3 Data for underlying data. (E) Zoomed-in fluorescence images of stage III Tg(Xla.Eef1a1:dclk2a-GFP) injected with Dextran Alexa 647 next to the GV (black dashed line) for marking nucleoplasm before and after GVBD. Top row, microtubules; bottom row, Dextran. The white asterisk demarcates [file pbio.3002146.s004.tif]

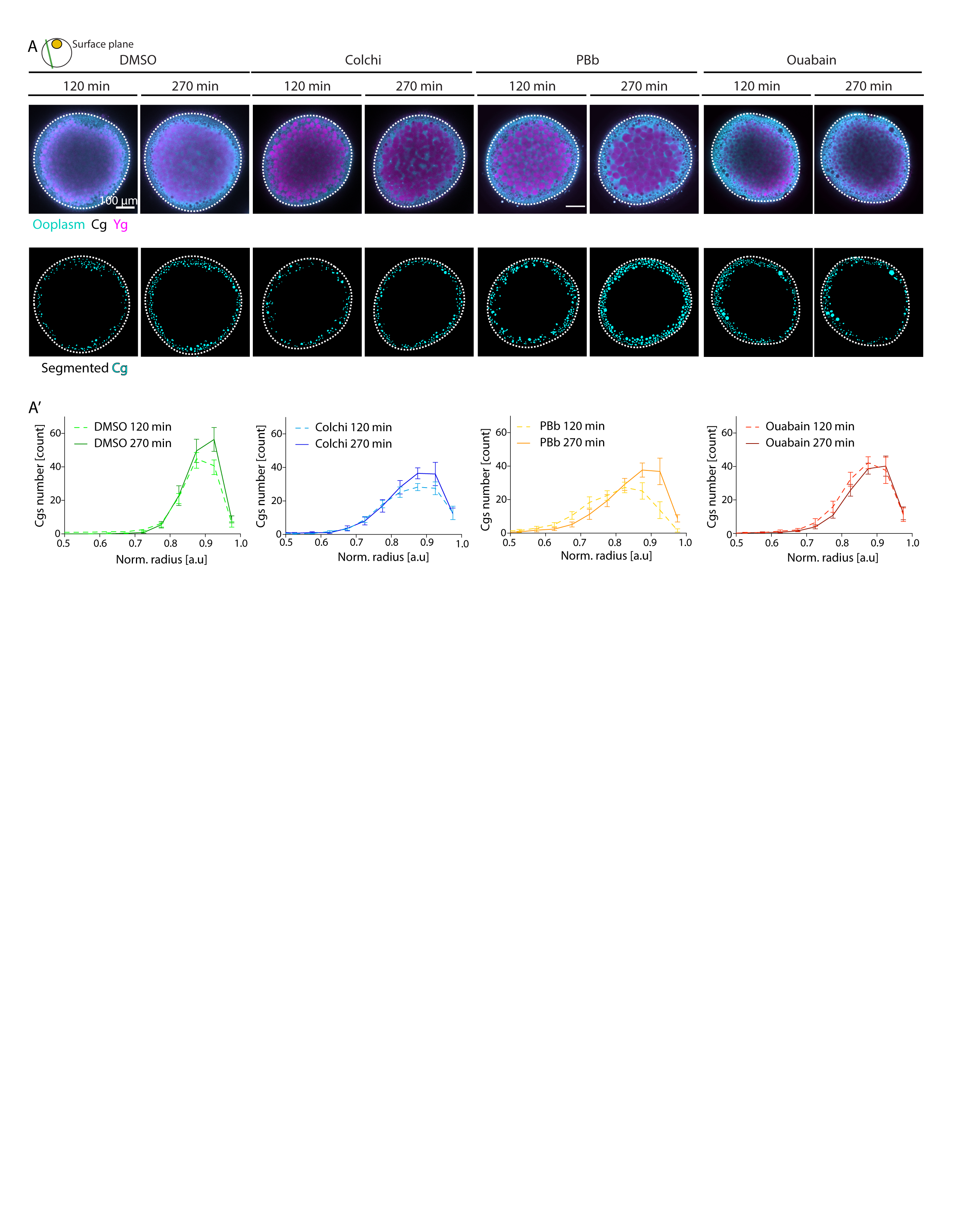

Supplement: S5 Fig — (A) Top row: Fluorescence images of stage III Tg(hsp:clip170-GFP) oocytes labelling ooplasm (cyan) exposed to Lysotracker to mark Ygs (magenta) 120 and 270 min after maturation induction for oocytes treated with DMSO, 200 μM Colchi, 100 μM PBb, or 100 μM Ouabain. Cgs (black) were identified by their exclusion of both Clip-170-GFP and Lysotracker. Bottom row: Segmented Cg obtained from the images in the top row. White dashed lines mark the oocyte outline. (A’) Cg density profile along the oocyte radius at 120 and 270 min after maturation onset for oocytes exposed to DMSO (green, N = 3 experiments, n = 11 oocytes), Colchi (blue, N = 3, n = 15), PBb (orange, N = 2, n = 12), or Ouabain (red, N = 3, n = 14). Normalized (norm.) radius of 0 and 1 correspond to the oocyte center and surface, respectively. Note that changes, but not the absolute values, in Cg distribution along the oocyte radius between 120 and 270 min reflect Cg translocation/movement during this time. See Table D in S4 Data for underlying data. Schematic in panel (A) demarcates the imaging plane used for obtaining the images in that panel. Error bars, SEM. Cg, cortical granule; Colchi, Colchicine; PBb, para-Nitroblebbistatin; Yg, yolk granule. (TIF) [file pbio.3002146.s005.tif]

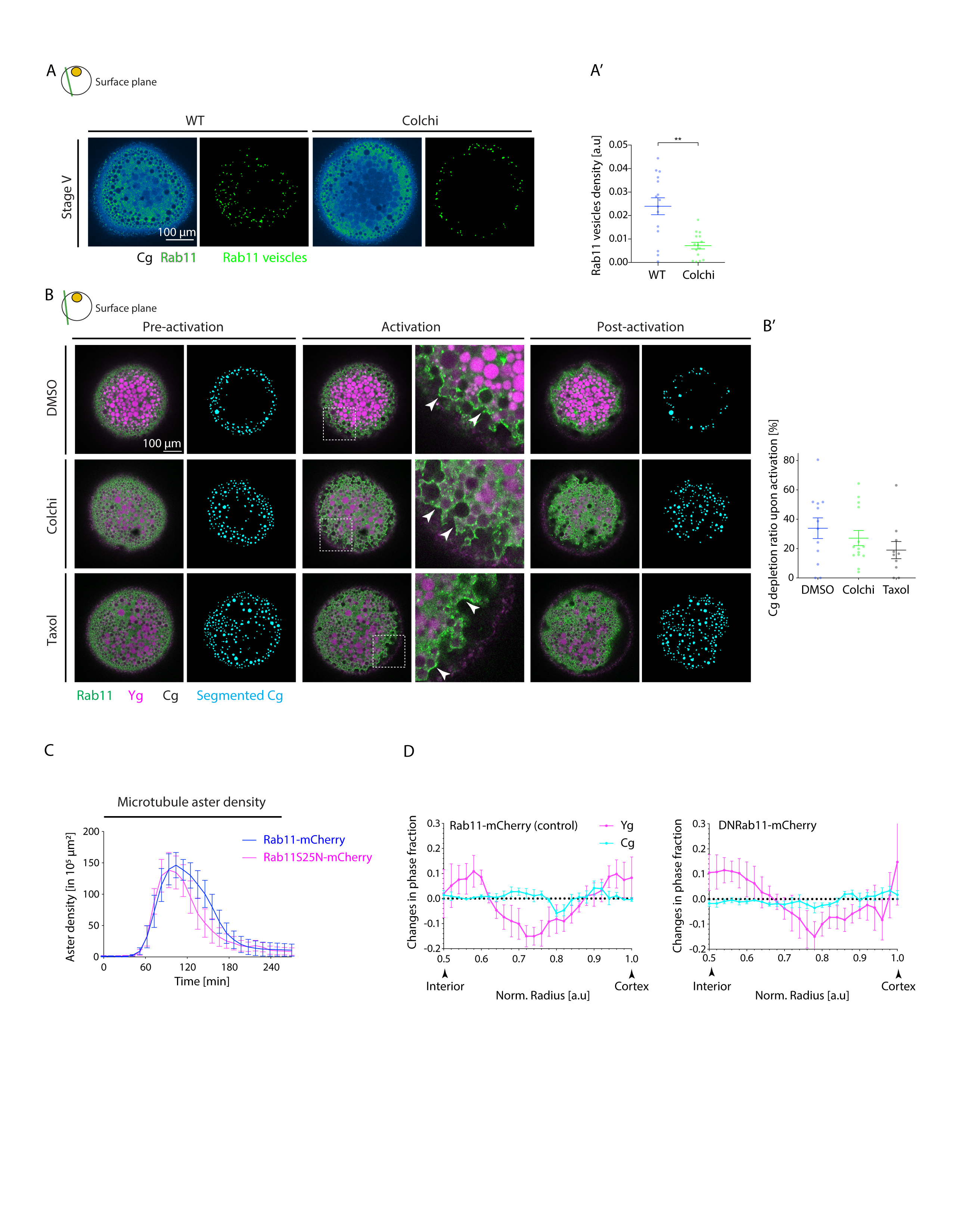

Supplement: S6 Fig — (A) Fluorescence images of stage-V Tg(actb2:Rab11a-NeonGreen) oocytes labelling Rab11+ vesicles. Left, WT oocytes; right, oocytes exposed to 200 μM Colchi. Images on the right of the fluorescence images show segmented Rab11+ vesicles obtained from the images on the left. (A’) Density of Rab11-positive vesicles, measured from the images in (A) for WT (blue, N = 2 experiments, n = 15 oocytes) and Colchi-treated oocytes (green, N = 2, n = 15). See Table E in S4 Data for underlying data. (B) Fluorescence images of Tg(actb2:Rab11a-NeonGreen) oocytes marking Rab11-positive vesicles (green) and exposed to Lysotracker to label Ygs (magenta) pre- (left), during (middle), and post-activation (right) with E3 medium and treated with DMSO (control, top), 200 μM Colchicine (Colchi, middle), or 50 μM Taxol (bottom). Cgs (black) are identified by their exclusion of Lysotracker and Rab11 signal. On the right of the fluorescence images (for pre- and post-activation) segmented Cg are shown. On the right of the fluorescence images (for activation) zoomed-in images are shown of the ROI demarcated by the white dashed boxes in the fluorescence images. Arrowheads mark exemplary Cg undergoing exocytosis. (B’) Cg depletion ratio upon egg activation for oocytes exposed to DMSO (blue, N = 3, n = 12), 200 μM Colchi (green, N = 3, n = 14) or 50 μM Taxol (black, N = 3, n = 10). See Table F in S4 Data for underlying data. (C) Microtubule aster density (count in 105 μm2) for oocytes injected with 350 pg Rab11-mcherry (blue, N = 3, n = 9) or Rab11S25N-mCherry (magenta; dominant negative, N = 3, n = 9) mRNA during oocyte maturation. See Table G in S4 Data for underlying data. (D) Changes in phase fractions of Yg (magenta) and Cg (cyan) for oocytes injected with 350 pg Rab11-mcherry (left, N = 2, n = 6) or Rab11S25N-mCherry (right, N = 2, n = 6) between 120 and 270 min after maturation onset. Normalized (norm) radii of 0.5 and 1 correspond to the oocyte interior and cortex, respectively. See Table H i [file pbio.3002146.s006.tif]

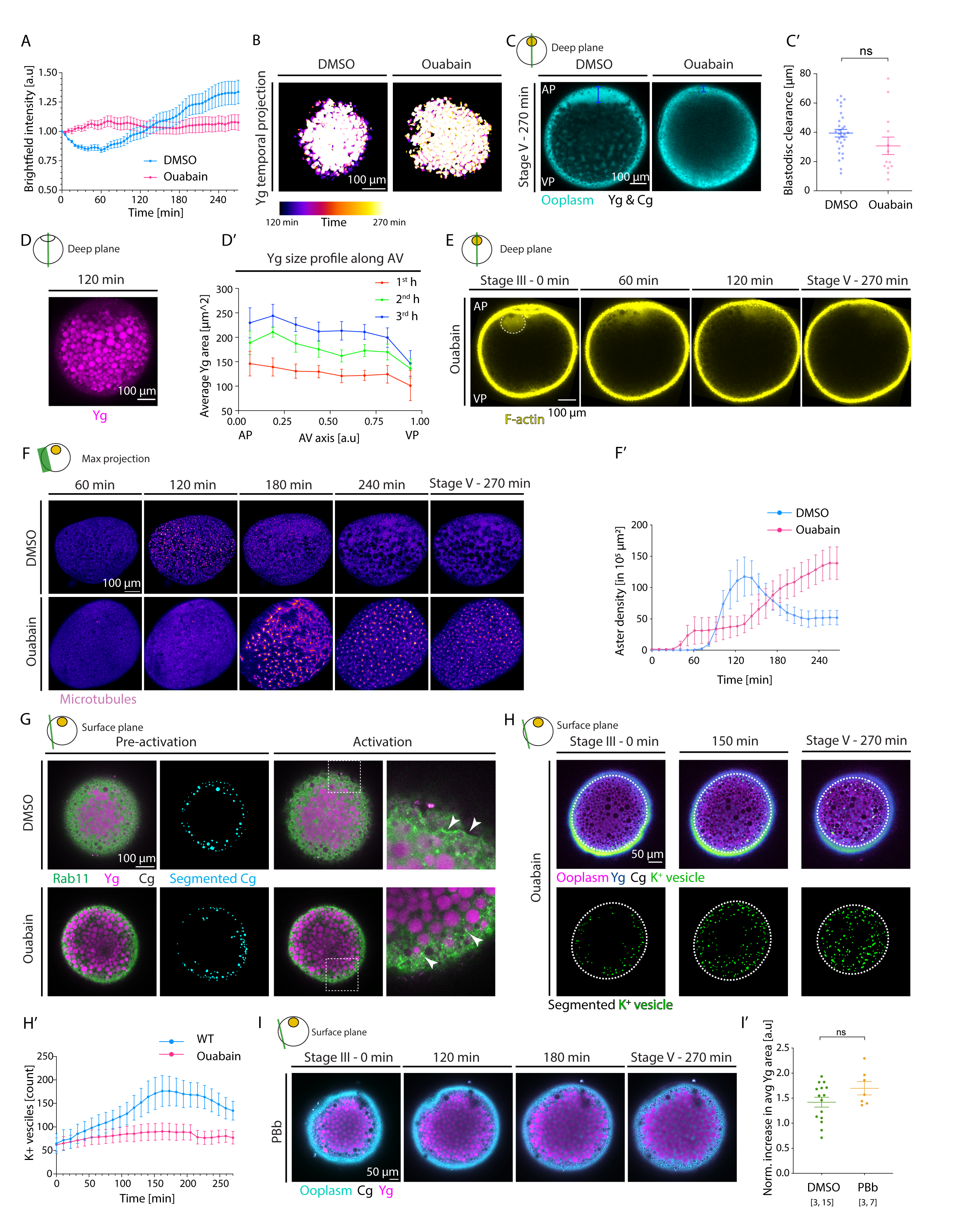

Supplement: S7 Fig — (A) Brightfield intensity of oocytes exposed to DMSO (control, blue, N = 3 experiments, n = 15 oocytes) or 100 μM Ouabain (magenta, N = 3, n = 14) within a 225 μm × 225 μm region in the oocyte center normalized to its value at stage III during oocyte maturation. See Table D in S5 Data for underlying data. (B) Temporal projection of segmented Ygs in oocytes exposed to DMSO (control, left) or Ouabain (right) (same oocytes as in Fig 5B) between 120 and 270 min after maturation onset. (C) Fluorescence images of stage V Tg(hsp:clip170-GFP) oocytes labeling the ooplasm exposed to DMSO (control, left) or 100 μM Ouabain (right). Ygs and Cgs are depicted by their exclusion of ooplasmic signal. Blue lines mark the blastodisc height as measured in (C’). (C’) Blastodisc clearance, measured as the height of blastodisc at the end of the maturation process, for oocytes exposed to DMSO (control, blue, N = 4, n = 29) or 100 μM Ouabain (magenta, N = 3, n = 13). See Table E in S5 Data for underlying data. (D) Maximum fluorescence intensity projection of oocytes exposed to Lysotracker to mark Yg 120 min after maturation onset. (D’) Yg area averaged for the first, second, and third hour (h) after maturation onset along the oocyte AV axis (N = 3, n = 8). See Table F in S5 Data for underlying data. (E) Fluorescence images of stage III Tg(actb1:Utr-GFP) oocytes labeling F-actin exposed to 100 μM Ouabain before (stage III) and 60, 120, and 270 min after maturation onset. The dashed line indicates the GV region. (F) Fluorescence images of stage III Tg(Xla.Eef1a1:dclk2a-GFP) oocytes labeling microtubules exposed to DMSO (top) or 100 μM Ouabain (bottom) at 60, 120, 180, 240, and 270 min after maturation onset. (F’) Microtubule aster density for oocytes exposed to DMSO (blue, N = 3, n = 9) or Ouabain (magenta, N = 2, n = 7) during oocyte maturation. See Table G in S5 Data for underlying data. (G) Fluorescence images of Tg(actb2:Rab11a-NeonGreen) oocytes marking Rab11-positive vesicles (green) a [file pbio.3002146.s007.tif]
